# Comparative Extraction of Phenolic Compounds from Olive Leaves Using a Sonotrode and an Ultrasonic Bath and the Evaluation of Both Antioxidant and Antimicrobial Activity

**DOI:** 10.3390/antiox11030558

**Published:** 2022-03-15

**Authors:** Beatriz Martín-García, Soumi De Montijo-Prieto, Maria Jiménez-Valera, Alegría Carrasco-Pancorbo, Alfonso Ruiz-Bravo, Vito Verardo, Ana María Gómez-Caravaca

**Affiliations:** 1 Department of Analytical Chemistry, Faculty of Sciences, University of Granada, Avd. Fuentenueva s/n, 18071 Granada, Spain; bea91mg@ugr.es (B.M.-G.); alegriac@ugr.es (A.C.-P.); anagomez@ugr.es (A.M.G.-C.); 2 Department of Nutrition and Food Science, Campus of Cartuja, University of Granada, 18071 Granada, Spain; 3 Department of Microbiology, Campus of Cartuja, University of Granada, 18071 Granada, Spain; soumidemontijop@ugr.es (S.D.M.-P.); mjvalera@ugr.es (M.J.-V.); aruizbr@ugr.es (A.R.-B.); 4 Biomedical Research Center, Institute of Nutrition and Food Technology ‘José Mataix’, University of Granada, Avda del Conocimiento sn., Armilla, 18100 Granada, Spain

**Keywords:** olive leaves, phenolic compounds, sonotrode, Box–Behnken, HPLC–MS, antimicrobial activity

## Abstract

A sonotrode ultrasound-assisted extraction of phenolic compounds from olive leaves has been developed using a Box–Behnken design to optimize the effects of solvent composition and ultrasound parameters. The determination of single phenolic compounds was performed by HPLC–MS and the highest recovery in total compounds, oleuropein and hydroxytyrosol was achieved using EtOH/H_2_O (55:45, *v*/*v*), 8 min and 100% of amplitude. The optimal conditions were applied on leaves from seven olive cultivars grown under the same conditions and the results were compared with those found by using a conventional ultrasonic bath, obtaining no statistical differences. Moreover, antioxidant activity by FRAP, DPPH and ABTS in these olive leaf extracts was evaluated and they exhibited a significant correlation with oleuropein and total phenolic content. All cultivars of olive leaf extracts were found to be active against *S. aureus* and methicillin-resistant *S. aureus* with minimum bactericidal concentration (MBC) values) that ranged from 5.5 to 22.5 mg mL^−1^. No extracts showed antimicrobial activity against *C. albicans*. The percentages of mycelium reduction in *B. cinerea* ranged from 2.2 and 18.1%. Therefore, sonotrode could be considered as an efficient and fast extraction technique that could be easily scaled-up at industrial level, thus allowing for olive leaves to be revalorized.

## 1. Introduction

Olive leaves represent about 10% of the total biomass of olives collected for the production of olive oil and around 25 kg of leaves are lost per olive tree during tree pruning [1]. Some of this by-product is used for animal food or bioenergy, whereas most of the olive leaves are discarded, thus causing both a high cost and a large environmental impact [2,3]. However, olive leaves contain phenolic compounds that possess several beneficial properties attributed in part to their antioxidant activity [4,5]. Therefore, this by-product could be used to obtain ingredients used in the production of nutraceuticals or functional foods.

Many factors, including the time of collection, cultivation zone, cultivar and agronomical conditions, affect the phenolic composition of olive leaves [2,6,7,8,9]. The main phenolic compounds in olive leaves are hydroxytyrosol, rutin, verbascoside, luteolin-7-glucoside, luteolin-4-O-glucoside, oleuropein, oleuropein aglycone and ligstroside aglycone [4,9,10]. In vitro and in vivo studies have reported that oleuropein and hydroxytyrosol derivatives possess a wide variety of biochemical and pharmacological properties such as antiproliferative, anti-inflammatory, antidiabetic, hypocholesterolemic and antimicrobial properties [10,11,12,13,14,15,16]. Therefore, these beneficial effects attributed to these bioactive compounds in the olive leaf extract explain the increasing interest shown by the pharmaceutical, cosmetic, nutraceutical and food industries. Specifically, the antimicrobial and antioxidant activities of these phenolic compounds present in olive leaves give them a potential use as natural additives/supplements [3].

The extraction process is an important step in order to obtain a high phenolic recovery from the samples. Maceration is a conventional extraction technique, which has long been used in the extraction of phenolic compounds in plants. Nevertheless, this technique requires high volumes of solvents, long extraction times and possesses low selectivity, low reproductive rates and low efficiency [17,18]. Nowadays, new efficient extraction processes such as microwave extraction, supercritical fluid extraction and pressurized liquid extraction have been used in the phenolic recovery from olive leaves [2,19,20,21]. These extraction techniques require short extraction times and low volumes of solvent in comparison with conventional ones. Nevertheless, most of these techniques operate at high pressures, which generate high energetic costs. For this reason, ultrasound assisted extraction (UAE) could be the best choice because it is an effective and low-cost extraction technique [22]. Two types of devices are used in ultrasound-assisted extraction: an ultrasonic bath and ultrasonic probe (sonotrode) equipment [23]. The ultrasonic bath is the most frequently used tool for phenolic extraction due to it being inexpensive and available, and allows the extraction of various samples simultaneously. However, by comparison with probe systems, it possesses a low reproducibility/production rate and a low power of ultrasound delivered directly to the sample and it is not practical at an industrial scale [23]. Indeed, the sonotrode system is more powerful in comparison with the ultrasonic bath because the ultrasonic intensity is delivered through a smaller surface (the tip of the probe) [24].

Considering the above-mentioned reasons, the purpose of this study was to evaluate the recovery in both complete and single phenolic compounds from olive leaves by high-performance liquid chromatography coupled with mass spectrometry (HPLC–MS) after the optimization of an ultrasonic probe (sonotrode) method. To achieve this, a response surface methodology (RSM) was performed to evaluate the extraction parameters: % EtOH/H_2_O (*v*/*v*), amplitude and extraction time with an experimental Box–Behnken design. The optimal conditions established were applied in seven olive leaf cultivars grown under the same conditions. In addition, a conventional ultrasonic bath extraction was carried out in order to compare the final phenolic concentration with that obtained by the optimized sonotrode extraction. Additionally, the antioxidant and antimicrobial potentials were assessed in the olive leaf extracts obtained at optimal sonotrode conditions.

## 2. Materials and Methods

### 2.1. Chemicals and Reagents

Ethanol, methanol and LC–MS grade acetronitrile were purchased from Fisher Scientific (Leicestershire, UK), and water was purified using a Milli-Q system (Millipore, Bedford, MA, USA). The acetic acid used was purchased from Fluka (Buchs, Switzerland). Standard compounds, including hydroxytyrosol, tyrosol, rutin, luteolin-7-glucoside, apigenin7-glucoside, luteolin and apigenin, were purchased from Sigma–Aldrich (Saint Louis, MO, USA), and oleuropein was purchased from Extrasynthèse (Lyon, France).

### 2.2. Samples

Olive leaves from cultivars ‘Arbequina’, ‘Arbosana’, ‘Changlot Real’, ‘Frantoio’, ‘Picual’, ‘Koroneiki’ and ‘Sikitita’ were collected from “IFAPA, Centro Alameda del Obispo” in Córdoba, Spain (37°51′36.5″ N, 4°47′53.7″ W). Samples were harvested in mid-February 2020 and all cultivars were grown under the same agronomic and environmental conditions in the same olive orchard. Olive leaves were air dried at room temperature. Subsequently, leaves were ground using IKA A 10 Basic Mill (Retsch GmbH, Haan, Germany) and the resulting powder was stored at −20 °C until the extraction.

### 2.3. Extraction of Phenolic Compounds from Olive Leaves by Sonotrode and Ultrasonic Bath Extraction

The extraction was achieved with a sonotrode UP400St (Hielscher Ultrasonics GmbH, Teltow, Germany). Using 100 mL of an EtOH/H_2_O mixture (1:400 (*w*/*v*)), 0.25 g of powdered olive leaves were extracted. The ultrasound amplitude, percentage of EtOH/H_2_O and extraction time were varied according to a Box–Behnken experimental design. 

The ultrasonic bath extraction of phenolic compounds was performed as described previously by Talhaoui et al. [2] with certain modifications. Briefly, powdered leaves (0.1 g) were extracted three times using 10 mL of EtOH/H_2_O (80:20, *v*/*v*) by using an ultrasonic bath (Bandelin, Sonorex, RK52, Berlin, Germany) operating at a frequency of 35 kHz for 20 min. Two replicates of each sample were processed.

After both kinds of extraction, the olive leaf extracts were centrifugated at 1000× *g* for 10 min, the supernatant was collected, evaporated and reconstituted in 5 mL of methanol/water (1:1, *v*/*v*). The final extracts were filtered through 0.2 μm nylon syringe filters and stored at −18 °C until the analyses.

### 2.4. Experimental Design

A Box–Behnken design with three variables was carried out to optimize the extraction parameters and to obtain the highest phenolic recovery from olive leaves. In this study, the three independent variables were amplitude (X_1_), % EtOH (X_2_) and time (X_3_), with three levels for each variable. The ranges for the established parameters were amplitude (20, 60 and 100%), % EtOH/H_2_O (30:70, 65:35 and 100:0, *v*/*v*) and extraction time (1, 5.5 and 10 min). Amplitude percentage refers to the percentage of maximum power used. The extraction time was limited to 10 min to avoid solvent evaporation because of the high temperatures generated and limiting the range of amplitude percentage [25]. The design consisted of 15 combinations including 3 center points (Table 1).

The response variables were fitted to a second-order polynomial model equation obtained by the response surface methodology (RSM) (Equation (1)):

(1) $$Y=\beta_{0}+\overset{3}{\underset{i=1}{\sum }}\beta_{i}X_{i}+\overset{3}{\underset{i=1}{\sum }}\beta_{\mathrm{ii}}X_{\mathrm{ii}}{}^{2}+\overset{2}{\underset{i=1}{\sum }}\overset{3}{\underset{j=i+1}{\sum }}\beta_{\mathrm{ii}}X_{i}X_{j}$$

where Y is the response variable, which is the sum of oleuropein, sum of hydroxytyrosol and total compounds (sum of phenolic compounds and elenolic acids) in ‘Koroneiki’ olive leaf cultivars obtained by HPLC–MS. Χi and Χj are the independent factors, whereas β_0_, β_i_, β_ii_ and β_ij_ are the regression coefficients of the model for the mean, linear, quadratic and interaction term calculated from the experimental results by the least squares method. Model building, experimental results and designs were processed using STATISTICA 8.0 (StatSoft, Tulsa, OK, USA).

### 2.5. Analysis of Phenolic Compounds by HPLC–MS

Analyses of the olive leaves phenolic compounds were carried out following the previously validated method of Talhaoui et al. [9] using an Agilent 1200 Series Rapid Resolution liquid chromatograph (Agilent Technologies, Palo Alto, CA, USA), which is comprised of a binary pump, degasser and auto sampler. Phenolic compounds were separated using a Poroshell 120 EC-C18 (4.6 × 100 mm, 2.7 µm) from Agilent Technologies, at 25 °C and a flow rate of 0.8 mL min^−1^.

Hydroxytyrosol, tyrosol, oleuropein, rutin, luteolin-7-glucoside, apigenin-7-glucoside and luteolin were the standard compounds used for the quantification of compounds in the olive leaf extracts. The calibration curves were prepared at seven concentration levels from the limit of quantification (LOQ) to 100 mg L^−1^. All calibration curves revealed a good linearity among different concentrations, and the determination coefficients were higher than 0.9947 in all cases. The method used for analysis showed a limit of detection (LOD) within the range 0.0061–0.2366 mg L^−1^ and the LOQ was within 0.0203–0.7888 mg L^−1^ (Table S1).

### 2.6. Antioxidant Capacity

Three different assays were used to determine the antioxidant capacity of the extracts obtained from olive leaf cultivars. Trolox (6-hydroxy-2,5,7,8-tetramethylchromen-2-carboxylic acid) was used as standard and the results were expressed in mg of trolox equivalents (TE) g^−1^ of dry weight. The calibration curve for the standard trolox was constructed by means of the absorbance vs. concentrations (1–1000 mg/L). The measurement of the absorbance for trolox was carried out according to the following antioxidant assay protocols using the same volume as for the extracts.

#### 2.6.1. DPPH Radical Scavenging

DPPH assay was performed according to a procedure described previously [26]. The absorbance at 517 nm at 25 °C after 30 min was measured when 0.1 mL of the samples (extract and trolox) were added to 2.9 mL of 100 µM DPPH (2,2-diphenyl-1-picrylhydrazyl) methanol/H_2_O 4/1 (*v*/*v*) solution.

#### 2.6.2. ABTS Cation Radical Scavenging

This assay was carried out following the method described by Re et al. [27]. The ABTS reagent was diluted with EtOH until reaching an absorbance of 0.7 ± 0.02 at 734 nm at 30 °C. The assay comprised the addition of 10 µL of extracts/trolox to 1 mL of diluted ABTS reagent and then measuring the decrease in absorbance after 10 min.

#### 2.6.3. Ferric Reducing Antioxidant Power (FRAP)

A FRAP assay was carried out according to a method previously described by Pulido et al. [28]. Briefly, 30 µL of sample (extracts/trolox) was diluted with 90 µL of water, which was added to 0.9 mL of FRAP reagent. The FRAP reagent was prepared by mixing 25 mL of 0.3 mM acetate buffer (pH 3.6); 2.5 mL of 10 mM of TPTZ (2,4,6-Tri(2-pyridyl)-1,3,5-triazine) in 40 mM HCl solution and 2.5 mL of 20 mM FeCl_3_·6H_2_O solution. The absorbance was measured at 595 nm after 30 min.

### 2.7. Antimicrobial Activity

#### 2.7.1. Test Microorganisms

The antimicrobial activity of the ethanol extracts (EtOH/H_2_O, 80:20, *v*/*v*) was tested against *Staphylococcus aureus* (*S. aureus*), methicillin-resistant *Staphylococcus aureus* (MRSA), *Escherichia coli* (*E. coli*), *Salmonella enterica* serovar Typhimurium (*S. Typhimurium*), *Listeria monocytogenes* (*L. monocytogenes*), *Candida albicans* (*C. albicans*) and *Botrytis cinerea* CECT 2100 (*B. cinerea*). The bacterial strains and *C. albicans* were stored as glycerol stocks and reactivated by incubation in tryptic soy agar (TSA) at 37 °C for 24 h. *B. cinerea* was maintained and grown at 25 °C in Sabouraud dextrose agar.

#### 2.7.2. Agar-Well Diffusion Method

Antimicrobial activity against bacteria and *C. albicans* was assessed following the method described by Hayes and Markovic with modifications, as follows: 15 mL of molten Mueller–Hinton agar were poured into sterile petri dishes and allowed to set to form a base layer [29]. Four stainless steel cylinders of 8 mm diameter were equidistantly placed over the base layer, and 10 mL of molten Mueller–Hinton agar containing the inoculum were poured over the surface of the base layer and left to set. For the preparation of the inoculum, cultures from the strains were suspended in the buffered saline solution until they reached a turbidity corresponding to 0.5 McFarland standard and were inoculated in molten Mueller–Hinton agar to obtain a final concentration of approximately 1 × 10^6^ CFU mL^−1^. After solidification of the upper layer, the cylinders were carefully removed and 120 μL of extracts were added into the resulting hole with concentrations between 87 and 94 mg mL^−1^. The antimicrobial activity controls used were as follows: 120 µL of ciprofloxacin 2 mg mL^−1^ for MRSA, 0.1 mg mL^−1^ for L. monocytogenes, 0.01 mg mL^−1^ for the rest of bacteria and ketoconazole 0.01 mg mL^−1^ for *C. albicans*. After incubation at 4 °C for 30 min to allow extracts to diffuse into the medium, the plates were incubated at 37 °C for 24 h. The inhibition zone diameters were measured (mm) and recorded as the mean ± standard deviation. Three replicates were carried out.

For *B. cinerea*, Sabouraud dextrose plates were prepared following the previous procedure but by testing one extract per plate. After 30 min incubation to allow the extract to diffuse, a 4-mm plug of mycelium was placed at a 3 cm distance from the extract. The plates were incubated for 7 days at 25 °C. The control consisted of Sabouraud dextrose plates inoculated with mycelium alone. The percentage of mycelium inhibition for each extract was calculated by measuring the area of fungal growth and comparing it to the control using the ImageJ 1.52 a software [30]. Three replicates were carried out.

#### 2.7.3. Determination of Minimum Inhibitory Concentration and Minimum Bactericidal Concentration

Minimum inhibitory concentration (MIC) values were assessed by the microdilution method following CLSI recommendations. Briefly, the extracts were diluted in Mueller–Hinton broth and two-fold dilutions series were prepared in 96-well plates with concentrations from 47 to 0.1 mg mL^−1^ 5 µL of strain suspensions corresponding to 0.5 McFarland standard were inoculated into wells containing 100 µL from each dilution to obtain a final concentration of 5 × 10^5^ cell mL^−1^. The microplates were incubated at 37 °C for 24 h. Assays were carried out in triplicate. The MIC was defined as the lowest concentration resulting in no visible growth of tested organisms. Minimum bactericidal concentration (MBC) values were obtained by growing 10 µL of each well into tryptic soy agar and incubating at 37 °C for 24 h. The MBC value was recognized as the lowest concentration that inhibited the growth of tested organisms. The results were expressed in mg mL^−1^.

## 3. Results and Discussion

### 3.1. Characterization of Phenolic and Other Compounds from Olive Leaf Extracts by HPLC–MS

Olive leaf extracts obtained by sonotrode and ultrasonic bath were analyzed by HPLC coupled to MS. Phenolic compounds present in the olive leaf extracts were identified by rendering their mass spectra and using the data reported in previous studies [9,13,31,32,33,34]. A total of 36 compounds were identified and quantified in olive leaf extracts obtained by sonotrode. The quantification of individual compounds in each experiment was carried out by using the calibration curve of different standards (Table S2).

Due to the numerous pharmacological properties shown by oleuropein and hydroxytyrosol, the authors decided to establish the best extraction conditions to obtain the highest recovery from these bioactive compounds.

### 3.2. Fitting the Model

The Box–Behnken experimental design was elaborated for the optimization of the sonotrode conditions, and considered experimental values obtained for the variable responses, as exhibited in Table 1. Regarding the experimental results, the sum of oleuropein isomers ranged from 13.6 mg g^−1^ d.w. in the run 1 (20% amplitude, 30% EtOH and 5.5 min) to 22.8 mg g^−1^ d.w. in the run 15 (60% amplitude, 65% EtOH and 5.5 min), whereas the sum of hydroxytyrosol isomers ranged from 0.64 mg g^−1^ d.w. in the run 10 (60% amplitude, 100% EtOH and 1 min) to 0.79 mg g^−1^ d.w. in the run 2 (100% amplitude, 30% EtOH and 5.5 min). Moreover, the content of total compounds ranged between 24.92 mg g^−1^ d.w. in run 10 (60% of amplitude, 100% EtOH and 10 min) and 34.15 mg g^−1^ d.w. in run 13 (60% amplitude, 65% amplitude and 5.5 min).

The evaluation of the model was carried out according to the significance of the regression coefficients. According with previous studies, the level of significance was α < 0.1 in order to increase the number for significant variables. The significant variables on the response variable for the sum of the oleuropein were the linear effect of amplitude (X_1_) (*p* = 0.004597) and its quadratic effect (X_11_) (*p* = 0.015641), % EtOH (X_2_) (*p* = 0.001780) and its quadratic effect (X_22_) (*p* = 0.002125), the cross effect between amplitude and % EtOH (X_12_) (*p* = 0.006317) and the linear effect of time (X_3_) (*p* = 0.084605). In addition, the significant effects for the sum of hydroxytyrosol were the intercept (X_0_) (*p* = 0.000010), amplitude (X_1_) (*p* = 0.028315), % EtOH (X_2_) (*p* = 0.027004) and its quadratic effect (X_22_) (*p* = 0.019649), the linear effect of time (X_3_) (*p* = 0.032025) and its quadratic (X_33_) (*p* = 0.039507), the cross effect between amplitude and % EtOH (*p* = 0.050152) and the cross effect between % EtOH with time (X_23_) (*p* = 0.058351). Finally, the significant variables on the response variable of total compounds were the intercept (X_0_) (*p* = 0.012806), the linear effect of amplitude (X_1_) (*p* = 0.020174), the linear effect of EtOH (X_2_) (*p* = 0.003687) and its quadratic effect (X_22_) (*p* = 0.003091), the linear effect of time (X_3_) (*p* = 0.062779) and its quadratic effect (X_33_) (*p* = 0.071892) and the cross effect between the amplitude and % EtOH (X_12_) (*p* = 0.019798).

An analysis of variance (ANOVA) with 95% confidence level was generated and the effect and regression coefficients of individual linear, quadratic and interaction terms were determined. ANOVA revealed that the models presented high correlation between independent factors and response variables with coefficients of determination (R^2^) between 0.89055 and 0.99282. The *p*-value for lack-of-fit was used to verify the adequacy of the model, which was non-significant (*p* > 0.05), thus, the model fits well (Table 2). Moreover, models were statistically acceptable since *p*-value was lower than 0.05 for all cases.

The regression coefficients of the models and the results of the analysis of variance (ANOVA) are shown in Table 2.

### 3.3. Analysis of Response Surfaces

**Figure 1:**
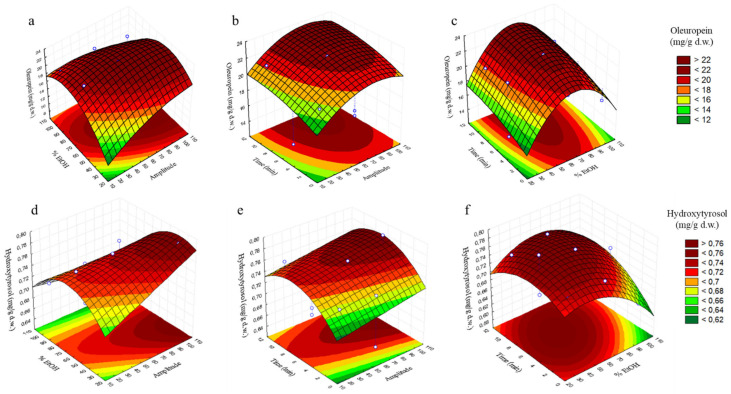
Response surface plots showing combined effects of process variables for oleuropein and hydroxytyrosol (mg/g d.w.): amplitude—% EtOH (**a**,**d**), amplitude—time (min) (**b**,**e**) and % EtOH—time (min) (**c**,**f**).

**Figure 2:**
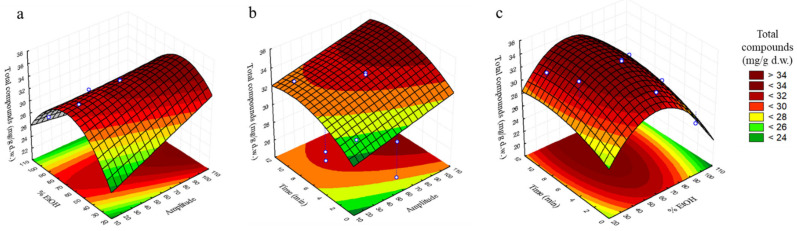
Response surface plots showing combined effects of process variables for total compounds (mg/g d.w.): amplitude—% EtOH (**a**), amplitude—time (min) (**b**) and % EtOH—time (min) (**c**).

In order to determine the optimal levels of independent variables for the extraction of the total content of phenolic compounds from olive leaves, response surfaces were plotted. Each pair of variables was depicted in three-dimensional surface plots, whereas the other variable was kept constant at central level. Figure 1a–f and Figure 2a–c are the three-dimensional plots showing the effects of amplitude (X_1_) and % EtOH (X_2_) (a), amplitude (X_1_) and time (X_2_) (b) and % EtOH (X_2_) and time (X_3_) (c) on the content of oleuropein, hydroxytyrosol and total compounds.

Figure 1a shows the maximum content of oleuropein in the range of 70–100% amplitude and 50–70% EtOH, whereas in Figure 1b, its maximum concentration is observed at 8–10 min and 60–100% amplitude and in Figure 1c, its maximum value shows at 8–10 min and 55–70% EtOH. Regarding hydroxytyrosol, in Figure 1d, its maximum content can be seen at 30–55% EtOH with the increasing in amplitude; in Figure 1e, an increase on the hydroxytyrosol content is shown with the rising of time and amplitude, which maximum content shows in the range of 6–9 min and 90–100% amplitude, whereas in Figure 1f, its maximum content shows at 3–10 min and 40–60% EtOH. Finally, concerning total phenolic compounds, an increase with the rising of % EtOH can be observed at a maximum amplitude value of 100% in Figure 2a, to arrive at its maximum value between 40–60% EtOH, from which the response starts to decrease. In Figure 2b, the maximum concentration of total compounds is observed at 8–10 min and 100% amplitude, and in Figure 2c, the maximum value on the response can be observed at 50–70% EtOH and 4–10 min.

### 3.4. Optimization of Sonotrode Parameters

The determination for the optimal conditions through the 3-D plots is the final step of the RSM. The optimal conditions to obtain the highest content of total phenolic compounds, oleuropein and hydroxytyrosol from olive leaves are shown in Table 3. Regarding the suggested models, the optimal conditions were 100% amplitude, EtOH/H_2_O (55:45, *v*/*v*) and 8 min to obtain as predictable values: 23 ± 2 mg g^−1^ d.w. of oleuropein, 0.8 ± 0.3 mg g^−1^ d.w. of hydroxytyrosol and 36 ± 5 mg g^−1^ d.w. of total phenolic compounds. The same optimal conditions were obtained for all responses; this fact suggests a positive correlation between the extraction of total compounds and the individual compounds oleuropein and hydroxytyrosol.

To verify the suitability of the model for total compounds, the predictable values were compared with the experimental values obtained at optimal conditions. The experimental values obtained by HPLC–MS were 24.6 ± 0.2 mg g^−1^ d.w. of oleuropein, 1.01 ± 0.02 mg g^−1^ d.w. of hydroxytyrosol and 40.9 ± 0.2 mg g^−1^ d.w. of total compounds. Analysis of the results revealed not significant differences between the theoretical and experimental data. Therefore, the models were considered suitable for these responses (Table 3). Optimal conditions for sonotrode extraction were compared with those obtained in previous studies in olive leaf samples. Martínez-Patiño et al. [25] reported the optimization of ethanol/water ratio (20, 50 and 80% EtOH), amplitude percentage (30, 50 and 70%) and ultra-sonication time (5, 10 and 15 min) on the responses of total phenolic content. This study reported a similar percentage of ethanol at 50% to obtain the highest phenolic content of 42 mg gallic acid eq g^−1^ d.w., whereas the amplitude and time (70% and 15 min) were different in comparison with our study [25]. Another study evaluated the effects of amplitude, % EtOH and time on total phenolic content (TPC), oleuropein content and total antioxidant activity of olive leaf extracts [35]. Vural et al. [35] reported the optimal conditions at 79.16% of amplitude, 73.40% EtOH and 12.90 min to obtain the highest total phenolic content and oleuropein content of 5.24 ± 0.08 mg g^−1^ d.w. and 2.22 ± 0.08 mg g^−1^ d.w., for which values were 87.19% and 90.97% lower than those obtained in our study, respectively. In addition, another study carried out the optimization of the effect on different modes of ultrasound operation (pulsed and continuous), liquid–solid (L–S) ratio and sonication time on the responses of oleuropein, verbascoside and luteolin-4′-O-glucoside, the amplitude and % EtOH were fixed at 70% of amplitude and 80% EtOH [36]. This study reported the optimum conditions as 10 cycles, liquid to solvent ratio 15:1 and 4 min to obtain the highest oleuropein content of 13.386 mg g^−1^ d.w., which was 45.6% lower than that obtained in the present study [36]. Da Rosa et al. [37] reported the extraction of phenolic compounds with an ultrasound system with a probe in ‘Arbequina’ olive leaf samples at the conditions for water, 29 min and 55% of amplitude to obtain 80.51 mg gallic acid eq g^−1^ d.w. The present study showed a shorter extraction time and higher amplitude value in comparison with these previous studies. This could be due to the fact that these studies did not evaluate a maximum value for amplitude in their design. However, the increase in amplitude led to an increase in the cavitation effects of the ultrasonics, which provides the formation and collapse of the cavitation bubbles during wave propagation. The implosion of the bubbles generates microjets and solvent flows, which in turn led to the cell rupture and mass transfer increasing the release of phenolic compounds from the matrix into the solvent [38].

In addition, these optimal conditions established in ultrasonic assisted extraction by sonotrode were applied to six other different olive leaf cultivars ‘Arbequina’, ‘Arbosana’, ‘Changlot Real’, ‘Frantoio’, ‘Picual’ and ‘Sikitita’, all grown under the same conditions. Concentrations of the individual phenolic compounds in these seven olive leaf cultivar extracts by sonotrode are reported in Table 4. In addition, the phenolic content found at optimal conditions by sonotrode was compared with the obtained by a conventional extraction by ultrasonic bath (Table S3). According to the results, the content of oleuropein, hydroxytyrosol and the total compounds obtained by UAE sonotrode and UAE bath did not show significant differences among them in all olive leaf cultivars. Therefore, the probe system has been shown to be an efficient extraction system because the direct contact with the sample allows the developing of a power-up to 100 times more than that provided in the ultrasonic bath, which leads in shorter extraction times when compared to the ultrasonic bath [24,39]. Therefore, the use of a probe system requires a lower extraction time of 8 min in comparison with the ultrasonic bath, which requires a total of 60 min carried out in three consecutive extractions to obtain the same recovery.

A one-way analysis of variance indicated significant differences among the seven cultivars (Table 4) (*p* < 0.05). The total content of compounds ranged from 28.1–49 mg g^−1^ olive leaves d.w. This range was in the same order of magnitude as the range of total phenolic content reported byTalhaoui et al. [40]. ‘Changlot Real’ was the cultivar that presented the highest total phenolic concentration, whereas ‘Picual’ was the cultivar that showed the lowest quantity of phenolic compounds. The total content of compounds in ‘Changlot Real’ was 42.7% higher than that obtained in ‘Picual’ [2]. In addition, according to the results, the total content of compounds obtained in ‘Arbequina’, ‘Arbosana’, ‘Frantoio’, ‘Koroneiki’ and ‘Sikitita’ did not show significant differences among them. These results are in concordance with Olmo-García et al. [41], who reported a similar content of phenolic compounds in ‘Arbequina’, ‘Frantoio’, ‘Koroneiki’ and ‘Picual’ (17.6, 28.0, 21.0 and 11.4 mg g^−1^ d.w.), Picual being the cultivar with the lowest phenolic content [41]. The total phenolic contents obtained in ‘Arbequina’, ‘Arbosana’ ‘Changlot Real’, ‘Koroneiki’, ‘Picual’ and ‘Sikitita’ were in the same range as the mean reported by Talhaoui et al. [2] (14.27–54.81 mg g^−1^ d.w.). Another study reported the total phenolic contents in ‘Arbequina’, ‘Sikitita’ and ‘Picual’ to be 30.74, 20.84 and 46.55% higher, respectively, than those reported in the present study (42, 41.26 and 28 mg g^−1^ d.w.) [9]. In addition, another study reported a total phenolic content in ‘Picual’, which was 66.3% higher than that obtained in our study, being similar for ‘Arbequina’ and ‘Sikitita’ (46.04 and 53.68 mg g^−1^ d.w.) [40]. These differences with previous studies could be due to different harvest times [2]. The sum of the oleuropein content ranged from 14.4 mg g^−1^ d.w. in ‘Picual’ to 34 mg g^−1^ d.w. in ‘Changlot Real’, displaying an increase of 57.6%. This oleuropein content range was in the same order of magnitude to the mean obtained by Talhaoui et al. [2] (10.38–45.35 mg g^−1^ d.w.). In addition, Talhaoui et al. [9] reported similar oleuropein contents in ‘Arbequina’, ‘Sikitita’ and ‘Picual’ (20.851, 20.849 and 21.653 mg g^−1^ d.w.). Another study reported a similar range for oleuropein content in ‘Picual’ (4.77–9.37 mg g^−1^ d.w.), ‘Arbequina’ (8.37–26.70 mg g^−1^ d.w.) and ‘Frantoio’ (6.35–30.17 mg g^−1^ d.w.) [42]. In addition, the content of oleuropein obtained for ‘Arbequina’ cultivar was 73.4% higher than the reported by Da Rosa et al. [37] by ultrasonic probe (6.91 mg g^−1^ d.w.). Furthermore, hydroxytyrosol content ranged from 0.37 mg g^−1^ d.w. in ‘Picual’ to 1.01 mg g^−1^ d.w. in ‘Koroneiki’, for which content was in the same range as that reported by Talhaoui et al. [2] (0.305–1.802 mg g^−1^ d.w.). In addition, the hydroxytyrosol content was also similar to that reported by Talhaoui et al. [9] in ‘Arbequina’, ‘Sikitita’ and ‘Picual’ cultivars. Another study reported a similar hydroxytyrosol content in Arbequina obtained by ultrasonic bath assisted with probe (0.547 mg g^−1^ d.w.) [37]. Therefore, according to these previous studies, there were differences in the phenolic content in the same olive leaf cultivars that could be due to the different harvest season, the climatic conditions, etc., because each cultivar possesses a resistance or tolerance to environmental conditions for each season [2].

### 3.5. Antioxidant Activity of Olive Leaves

Results for antioxidant activities in olive leaves are presented in Table 5. DPPH, ABTS and FRAP assays were carried out. DPPH and ABTS neutralized the two radicals (ABTS^•+^ and DPPH^•^) either by direct reduction via electron transfers or by radical quenching via hydrogen atom. Furthermore, the FRAP value measures the reduction in the ferric ion (Fe^3+^) to the ferrous ion (Fe^2+^) by donor electrons in the sample [43].

The experimental antioxidant activity of olive leaf extracts ranged from 33.03 mg of TE g^−1^ d.w in Picual to 46.8 mg of TE g^−1^ d.w. in the DPPH assay; and between 28.12–35.7 mg of TE g^−1^ d.w. in the ABTS assay and 37.17–53.87 mg of TE g^−1^ d.w. in the FRAP assays. According to the results, the highest antioxidant activity was obtained in ‘Changlot Real’, which was 29.42%, 15.63% and 27.16% higher than the lowest values presented in ‘Picual’ by DPPH, ABTS and FRAP, whereas the rest of the cultivars presented a similar antioxidant activity among them. Therefore, these results for antioxidant activities were related to the total phenolic content obtained by HPLC–MS. In addition, these values obtained by DPPH, ABTS and FRAP assays were in the same order of magnitude than those reported in a previous study, which were 42.5, 95.9 and 49.7 mg TE g^−1^ d.w. obtained in olive leaf extracts under the optimal conditions for sonotrode ultrasonic assisted extraction of 70% amplitude, 50% EtOH and 15 min [25].

The statistical correlation between phenolic compounds and antioxidant activities appears in Table 6. DPPH exhibit a significant positive correlation with oleoside, secologanoside isomer a and b, tyrosol glucoside, tyrosol, elenolic acid glucoside isomer b, luteolin glucoside isomer c, oleuropein isomer a and b, oleuropein/oleuroside and ligstroside.

Furthermore, ABTS exhibited a high significant positive correlation with the following free phenolic compounds: hydroxytyrosol–hexose isomer a, oleoside, hydroxytyrosol, secologanoside isomer a and b, elenolic acid glucoside isomer b, demethyloleuropein, verbascoside, apigenin-7-glucoside, oleuropein diglucoside isomer c, chrysoeriol-7-O-glucoside and oleuropein isomer a.

The FRAP assay showed a significant positive correlation with hydroxytyrosol–hexose isomer a, oleoside, secologanoside isomer a and b, caffeoylglucoside, elenolic acid glucoside isomer b, demethyloleuropein, hydroxyoleuropein isomer a, verbascoside, apigenin-7-O-glucoside, oleuropein diglucoside isomer c and oleuropein isomer a.

According to these results, the most abundant phenolic compounds: secologanoside isomer a and b, elenolic acid glucoside isomer b and oleuropein isomer a, possess a high correlation with the antioxidant activities, with the exception of luteolin glucoside that did not show a correlation with ABTS, DPPH and FRAP. In addition, DPPH, ABTS and FRAP have shown a high correlation with the sum of oleuropein (r = 0.77, r = 0.66 and r = 0.77) and total compounds (r = 0.82, r = 0.65 and r = 0.70). These results agree with a study that found significant correlations between TEAC, FRAP and DPPH with oleuropein (r = 0.664, r = 0.836 and r = −0.674) and with the total phenolic content (r = 0.746, r = 0.885, r = −0.824) [44]. In addition, another study reported that TPC correlates strongly with all performed in vitro methods except for the ABTS assay. Therefore, differences in correlations between individual phenolic compounds and antioxidant activities by different assays could be explained by different responses of phenolic compounds to different antioxidant reaction mechanisms [45].

### 3.6. Antimicrobial Activity of Olive Leaves

The olive leaf extracts were assessed for antimicrobial activity against *S. aureus*, MRSA, *L. monocytogenes* (Gram +), *E. coli*, *S. Typhimurium* (Gram -), *C. albicans* and the grey mold disease agent *B. cinerea* (fungi). Most of these microorganisms have been implicated as causal agents of foodborne-disease outbreaks and food quality degradation [46,47,48,49]. All the cultivars of olive extracts showed antibacterial effects with zones of inhibition ranging from 10 to 20.3 mm (Table S4). ‘Changlot Real’, ‘Frantoio’ and ‘Koroneiki’ cultivars showed the highest inhibition halos against *S. aureus* and MRSA. No extracts showed antimicrobial activity against *C. albicans*. As the MIC values could not be determined because the color of extracts masked the visible growth of the tested microorganisms, the MBC was determined by the culturing of all wells in TSA plates (Table 7). MBC values were found between 5.5 to 45 mg mL^−1^. ‘Frantoio’ leaf extract exhibited the highest antibacterial activity with an MBC value of 5.5 mg mL^−1^ against *S. aureus* and MRSA. The antimicrobial activity of olive leaf extracts has been demonstrated against a wide group of microorganisms. Testa et al., 2019 found inhibition zones between 12 and 17 mm, and MIC values ranging from2 to 5 mg mL^−1^ of olive leaf extract ‘Gentile di Larino’ cultivar against spoilage bacterial strains [50]. Although our extracts showed antibacterial activity at similar concentrations against *S. aureus* and MRSA, they showed activity against *E. coli*, *S. Typhimurium* and *L. monocytogenes* at higher concentrations. Olive leaf extracts have been found to be active against oral pathogens, including *C. albicans* [51] with MIC and MBC values ranging from 0.07 to 10 mg mL^−1^ and 0.60 to 10 mg mL^−1^, respectively. None of our extracts showed activity against *C. albicans*. In agreement with our results, Sudjana et al. [52] reported a non-broad-spectrum activity of the extracts, showing activity only against *H. pylori*, *C. jejuni*, *S. aureus* and MRSA, whereas some studies have reported a broad antimicrobial activity of olive leaf extracts ‘Cobrançosa’ cultivar against causal agents of human intestinal and respiratory tract infections in a concentration-dependent manner [53].

*B. cinerea* causes severe damage to agriculture in pre-and post-harvest and important losses in more than 200 crop species worldwide [49]. All cultivars showed lower inhibitory effects against *B. cinerea*. ‘Arbosana’, ‘Picual’ and ‘Sikitita’ cultivars showed the best percentages of mycelium reduction with 15, 15.4 and 18.1%, respectively, whereas ‘Changlot real’, ‘Koroneiki’, ‘Frantoio’ and ‘Arbequina’ cultivars inhibited 10.4, 10.2, 6.5 and 2.2%.

Although the MIC values were too high to be considered effective, the activity against pathogenic bacteria suggests the presence of potentially interesting bioactive compounds that conditioned the observed antimicrobial effect. The antimicrobial activity of phenolic compounds present in the plant extracts is well known [54,55]. Oleuropein is the main phenolic compound in olive leaf extracts with reported antioxidant and antimicrobial properties [16,56]. The high concentration of phenolic compounds and derivatives found might contribute to their antimicrobial properties. However, it is important to underline that the antimicrobial activity of a complex extract is not only related to the phenolic composition, but also to the synergisms/antagonisms among the compounds. In fact, other authors [3] have noticed that the antimicrobial activity of phenolic compounds is related to the denaturation of protein, inhibition of bacterial growth and enhancing of the permeability in cell membranes. Thus, further studies will be necessary to understand the possible mechanisms.

## 4. Conclusions

In conclusion, the phenolic content obtained by sonotrode ultrasound-assisted extraction at the optimum conditions: ethanol/water (55:45, *v*/*v*), 100% amplitude and 8 min, did not show significant differences with the presented method in a conventional extraction by ultrasonic bath. Therefore, the sonotrode ultrasound-assisted extraction, which can be implemented at an industrial scale, is an effective extraction technique to obtain olive leaf extracts enriched in phenolic compounds in shorter extraction times than conventional extraction. In addition, DPPH, ABTS and FRAP have exhibited a significative correlation with the content of oleuropein and total compounds of the extracts; most of them were highly active against methicillin-resistant *Staphylococcus aureus* and able to produce mycelium reduction in *Botrytis cinerea*.

## Figures and Tables

**Table 1:** Box–Behnken design with sonotrode parameters and values for dependent variables obtained (oleuropein, hydroxytyrosol and total compounds) quantified by HPLC–MS ‘Koroneiki’ in olive leaves. Results are expressed as mean ± standard deviation in mg g^−1^ dry matter of olive leaves. Different letters in the same column indicate significantly different values (*p* < 0.05).

Runs	X_1_	X_2_	X_3_	Oleuropein (mg g^−1^ d.w.)	Hydroxytyrosol (mg g^−1^ d.w.)	Total Compounds (mg g^−1^ d.w.)
1	20 (38 W)	30	5.5	13.6 ± 0.1 ^i^	0.697 ± 0.006 ^b,c^	26.5 ± 0.6 ^e,f^
2	100 (149 W)	30	5.5	21.0 ± 0.4 ^c,d,e^	0.79 ± 0.02 ^a^	33.7 ± 0.8 ^a^
3	20 (29 W)	100	5.5	19.0 ± 0.4 ^f,g^	0.709 ± 0.002 ^a,b,c^	27.5 ± 0.5 ^d,e^
4	100 (126 W)	100	5.5	19.13 ± 0.04 ^f,g^	0.73 ± 0.03 ^a,b^	28.31 ± 0.06 ^d^
5	20 (36 W)	65	1	20.10 ± 0.05 ^e,f^	0.736 ± 0.006 ^a,b^	31.20 ± 0.05 ^c^
6	100 (136 W)	65	1	21.2 ± 0.5 ^b,c,d,e^	0.72 ± 0.01 ^a,b,c^	32.5 ± 0.5 ^a,b,c^
7	20 (37 W)	65	10	21.47 ± 0.01 ^b,c,d^	0.76 ± 0.02 ^a,b^	33.0 ± 0.3 ^a,b^
8	100 (140 W)	65	10	21.8 ± 0.2 ^a,b,c^	0.746 ± 0.008 ^a,b^	33.0 ± 0.3 ^a,b^
9	60 (89 W)	30	1	18.1 ± 0.3 ^g,h^	0.73 ± 0.03 ^b^	28.9 ± 0.5 ^d^
10	60 (88 W)	100	1	17.5 ± 0.3 ^h^	0.64 ± 0.01 ^c^	24.92 ± 0.06 ^f^
11	60 (87 W)	30	10	20.32 ± 0.07 ^e^	0.75 ± 0.05 ^a,b^	31.82 ± 0.08 ^c^
12	60 (85 W)	100	10	20.4 ± 0.4 ^d,e^	0.72 ± 0.02 ^a,b,c^	29.2 ± 0.5 ^d^
13	60 (86 W)	65	5.5	22.771 ± 0.007 ^a^	0.76 ± 0.02 ^a,b^	34.153 ± 0.001 ^a^
14	60 (87 W)	65	5.5	22.30 ± 0.02 ^a,b^	0.768 ± 0.001 ^a,b^	33.29 ± 0.4 ^a,b^
15	60 (85 W)	65	5.5	22.8 ± 0.5 ^a^	0.75 ± 0.03 ^a,b^	34.0 ± 0.7 ^a^

X_1_: amplitude, X_2_: %EtOH (*v*/*v*), X_3_: time (min).

**Table 2:** Regression coefficients and analysis of variance (ANOVA) of the models for the variables of response of oleuropein, hydroxytyrosol and total compounds.

	Responses		
Regression Coefficients	Oleuropein	Hydroxytyrosol	Total Compounds
β_0_	−0.227440	0.414128 *	11.80061 *
Linear			
β_1_	0.207086 *	0.005551 *	0.15374 *
β_2_	0.428329 *	0.009575 *	0.46774 *
β_3_	0.367820 **	0.004871 *	0.68413 **
Cross product			
β_12_	−0.001293 *	−0.000154 **	−0.00114 *
β_13_	−0.001110	0.000153	−0.00190
β_23_	0.001030	0.000098 **	0.00225
Quadratic			
β_11_	−0.000743 *	−0.000014	−0.00034
β_22_	−0.002661 *	−0.000069 *	−0.00347 *
β_33_	−0.015611	−0.000981 *	−0.04123 **
Adequacy of the model			
R^2^	0.89055	0.99282	0.92365
*p* (model)	0.046761	0.011786	0.008413
*p* (lack of fit)	0.055317	0.076639	0.103196

* Significant at α < 0.05, ** Significant at α < 0.1.

**Table 3:** Optimal conditions for oleuropein, hydroxytyrosol and total compounds for sonotrode UAE. N.S.: no significant differences.

Optimal Conditions	Oleuropein	Hydroxytyrosol	Total Compounds
Amplitude (%) (Power)	100 (151 W)	100 (151 W)	100 (151 W)
EtOH (% (*v*/*v*))	55	55	55
Time (min)	8	8	8
Predicted (mg g^−1^ d.w.)	23 ± 2	0.8 ± 0.3	36 ± 5
Experimental (mg g^−1^ d.w.)	24.6 ± 0.2	1.01 ± 0.02	40.9 ± 0.2
Significant differences	N.S.	N.S.	N.S.

**Table 4:** Quantification of phenolic compounds obtained at optimum sonotrode conditions in different olive leaf cultivars. Results are expressed as mean ± standard deviation in mg g^−1^ dry matter of olive leaves. Different letters in the same line indicate significant differences among the cultivars.

Phenolic Compound	‘Arbequina’	‘Arbosana’	‘Changlot Real’	‘Frantoio’	‘Koroneiki’	‘Picual’	‘Sikitita’
Hydroxytyrosol–hexose isomer a	0.0081 ±0.0003 ^b^	0.0080 ± 0.0005 ^b^	0.0079 ± 0.0003 ^b^	0.00497 ± 0.00004 ^c^	0.0048 ± 0.0005 ^c^	0.0070 ± 0.0002 ^b^	0.00938 ± 0.00003 ^a^
Oleoside	0.5751 ± 0.0007 ^b^	0.49 ± 0.01 ^b^	0.71 ± 0.05 ^a^	0.47 ± 0.06 ^b^	0.48 ± 0.01 ^b^	0.27 ± 0.02 ^c^	0.510 ± 0.003 ^b^
Hydroxytyrosol–hexose isomer b	0.61 ± 0.02 ^d^	0.46 ± 0.01 ^e^	0.359 ± 0.003 ^f^	0.742 ± 0.001 ^c^	0.90 ± 0.02 ^a^	0.274 ± 0.009 ^g^	0.811 ± 0.008 ^b^
Hydroxytyrosol	0.112 ± 0.003 ^b^	0.091 ± 0.004 ^c^	0.0252 ± 0.0004 ^d^	0.125 ± 0.003 ^a^	0.1031 ± 0.0004 ^b^	0.089 ± 0.005 ^c^	0.107 ± 0.002 ^b^
Secologanoside isomer a	5.0 ± 0.1 ^b^	3.76 ± 0.06 ^c^	5.9 ± 0.2 ^a^	2.0 ± 0.2 ^d^	3.2 ± 0.2 ^c^	1.8 ± 0.2 ^d^	5.9 ± 0.3 ^a^
Tyrosol glucoside	0.156 ± 0.002 ^c^	0.066 ± 0.001 ^d^	0.136 ± 0.004 ^c^	0.081 ± 0.005 ^d^	0.196 ± 0.007 ^b^	0.016 ± 0.001	0.30 ± 0.01 ^a^
Caffeoyl glucoside	0.26 ± 0.01 ^b^	0.32 ± 0.01 ^a^	0.134 ± 0.002 ^c^	0.0168 ±0.0005 ^d^	0.039 ± 0.007 ^d^	0.020 ± 0.003 ^d^	0.044 ± 0.005 ^d^
Tyrosol	0.012 ± 0.003 ^a^	0.00439 ± 0.00005 ^c^	0.005 ± 0.001 ^c^	0.005 ± 0.001 ^c^	0.007 ± 0.001 ^b^	0.0016 ± 0.0003 ^c^	0.014 ± 0.002 ^a,b^
Elenolic acid glucoside isomer a	0.249 ± 0.009 ^b^	0.389 ± 0.008 ^a^	0.144 ± 0.007 ^d^	0.10 ± 0.01 ^e^	0.21 ± 0.01 ^c^	0.094 ± 0.005 ^e,f^	0.064 ± 0.003 ^f^
Secologanoside isomer b	0.99 ± 0.09 ^c,d^	1.12 ± 0.03 ^c^	0.78 ± 0.01 ^d^	2.64 ± 0.01 ^a^	2.149 ± 0.004 ^b^	2.6 ± 0.2 ^a^	0.87 ± 0.03 ^c,d^
Elenolic acid glucoside isomer b	0.737 ± 0.007 ^c^	0.86 ± 0.01 ^b^	0.780 ± 0.006 ^b,c^	1.02 ± 0.07 ^a^	0.94 ± 0.06 ^a^	0.907 ± 0.007 ^a^	0.685 ± 0.007 c
Oleuropein aglycon	0.74 ± 0.05 ^d^	1.6 ± 0.1 ^b^	1.7 ± 0.1 ^b^	1.20 ± 0.08 ^c^	2.7147 ± 0.0002 ^a^	1.63 ± 0.02 ^b^	1.0 ± 0.04 ^c,d^
Elenolic acid glucoside isomer c	0.26 ± 0.02 ^d^	0.36 ± 0.03 ^c,d^	0.92 ± 0.04 ^a^	0.40 ± 0.03 ^c^	0.53 ± 0.02 ^b^	0.87 ± 0.03 ^a^	0.65 ± 0.02 ^b^
Luteolin diglucoside	0.0279 ± 0.0004 ^a^	0.029 ± 0.002 ^a^	0.00792 ± 0.00005 ^d^	0.0091 ± 0.0006 ^c,d^	0.013 ± 0.002 ^b,c,d^	0.014 ± 0.001 ^b,c^	0.0180 ± 0.0009 ^b,c^
Elenolic acid glucoside isomer d	0.146 ± 0.006 ^c,d^	0.164 ± 0.004 ^b,c,d^	0.1313 ± 0.0002 ^d^	0.166 ± 0.002 ^b,c,d^	0.202 ± 0.008 ^b^	0.181 ± 0.002 ^b,c^	0.26 ± 0.03 ^a^
Demethyloleuropein	0.36 ± 0.01 ^b^	0.200 ± 0.003 ^c^	0.17 ± 0.01 ^c,d^	0.417 ± 0.005 ^a^	0.37 ± 0.01 ^b^	0.37 ± 0.02 ^b^	0.134 ± 0.005 ^d^
Hydroxyoleuropein isomer a	0.2237 ± 0.0008 ^c^	0.215 ± 0.002 ^c^	0.394 ± 0.001 ^b^	0.45 ± 0.03 ^b^	0.40 ± 0.02 ^b^	0.66 ± 0.05 ^a^	0.27 ± 0.01 ^c^
Rutin	0.391 ± 0.005 ^b^	0.924 ± 0.005 ^a^	0.131 ± 0.006 ^d^	0.28 ± 0.01 ^c^	0.46 ± 0.05 ^b^	0.109 ± 0.004 ^d^	0.29 ± 0.01 c
Luteolin rutinoside	0.0633 ± 0.0004 ^a^	0.064 ± 0.002 ^a^	0.0082 ± 0.0006 ^e^	0.0181 ± 0.0003 ^d^	0.038 ± 0.002 ^b^	0.0207 ± 0.0008 ^d^	0.031 ± 0.001 ^c^
Luteolin glucoside isomer a	3.0 ± 0.2 ^b^	3.8 ± 0.1 ^a^	1.49 ± 0.06 ^d^	2.017 ± 0.008 ^c^	1.74 ± 0.03 ^c,d^	1.42 ± 0.08 ^d^	2.05 ± 0.07 ^c^
Verbascoside	0.0129 ± 0.0001 ^a^	0.0108 ± 0.0006 ^b^	0.0107 ± 0.0006 ^b^	0.0053 ± 0.0006 ^c^	0.0054 ± 0.0004 ^c^	0.0066 ± 0.0002 ^c^	0.0092 ± 0.0004 ^b^
Hydroxyoleuropein isomer b	0.08 ± 0.02 ^b^	0.240 ± 0.006 ^a^	0.11 ± 0.02 ^b^	0.011 ± 0.002 ^c^	0.0166 ± 0.0009 ^c^	0.070 ± 0.001 ^b^	0.017 ± 0.006 ^c^
Apigenin rutinoside	0.0146 ± 0.0002 ^c^	0.027 ± 0.001 ^a^	0.0172 ± 0.0006 ^b^	0.0116 ± 0.0009 ^d^	0.0157 ± 0.0004 ^b,^	0.01656 ± 0.00005 ^b,c^	0.01631 ± 0.00004 ^b,c^
Oleuropein diglucoside isomer a	0.01441 ± 0.00008 ^b^	0.0129 ± 0.0002 ^b^	0.01445 ± 0.00003 ^b^	0.015 ± 0.006 ^a^	0.0135 ± 0.0004 ^b^	0.0108 ± 0.0006 ^c^	0.0136 ± 0.0006 ^b^
Apigenin-7-glucoside	0.054 ± 0.002 ^f^	0.158 ± 0.002 ^b^	0.182 ± 0.006 ^a^	0.0994 ± 0.0004 ^c^	0.064 ± 0.002 ^e,f^	0.06735 ± 0.00002 ^e^	0.084 ± 0.001 ^d^
Oleuropein diglucoside isomer b	0.017 ± 0.001 ^a,b^	0.017 ± 0.004 ^a,b^	0.013 ± 0.002 ^a,b^	0.0113 ± 0.0009 ^b,b^	0.021 ± 0.002 ^a^	0.012 ± 0.002 ^a,b^	0.018 ± 0.003 ^a,b^
Luteolin glucoside isomer b	1.11 ± 0.01 ^a^	1.19 ± 0.05 ^a^	0.60 ± 0.03 ^c^	0.81 ± 0.01 ^b^	0.68 ± 0.01 ^c^	0.85 ± 0.01 ^b^	0.90 ± 0.02 ^b^
Oleuropein diglucoside isomer c	0.021 ± 0.002 ^c,d^	0.029 ± 0.003 ^b,c^	0.043 ± 0.003 ^a^	0.01496 ± 0.00007 ^d,e^	0.0391 ± 0.0003 ^a,b^	0.00959 ± 0.00004 ^e^	0.034 ± 0.005 ^a,b^
Chrysoeriol-7-O-glucoside	0.0316 ± 0.0003 ^a,b^	0.0192 ± 0.0007 ^d^	0.0179 ± 0.0005 ^d^	0.0345 ± 0.0001 ^a^	0.029 ± 0.002 ^b^	0.0259 ± 0.0003 ^c^	0.0303 ± 0.0002 ^b^
Luteolin glucoside isomer c	0.155 ± 0.007 ^b,c^	0.219 ± 0.002 ^a^	0.075 ± 0.001 ^d,e^	0.06 ± 0.01 ^e^	0.19 ± 0.01 ^a,b^	0.21 ± 0.02 ^a^	0.115 ± 0.008 ^c,d^
Oleuropein isomer a	24 ± 1 ^b^	21.4 ± 0.5 ^b^	32 ± 1 ^a^	21.8 ± 0.3 ^b^	22.5 ± 0.5 ^b^	13.7 ± 0.3 ^c^	23.1 ± 0.3 ^b^
Oleuropein isomer b	0.430 ± 0.004 ^a^	0.38 ± 0.09 ^a^	0.49 ± 0.02 ^a^	0.38 ± 0.01 ^a^	0.6 ± 0.1 ^a^	0.075 ± 0.009 ^b^	0.495 ± 0.005 ^a^
Oleuropein/Oleuroside	1.54 ± 0.02 ^a^	1.3 ± 0.2 ^a^	1.47 ± 0.07 ^a^	1.382 ± 0.003 ^a^	1.5 ± 0.1 ^a^	0.59 ± 0.02 ^b^	1.58 ± 0.07 ^a^
Ligstroside aglycone	0.51 ± 0.02 ^c^	0.624 ± 0.003 ^b^	0.302 ± 0.003 ^d^	0.65 ± 0.02 ^b^	0.075 ± 0.004 ^e^	0.76 ± 0.02 ^a^	0.335 ± 0.003 ^d^
Ligstroside	0.5202 ± 0.0008 ^a^	0.29 ± 0.01 ^d,e^	0.27 ± 0.02 ^e^	0.33 ± 0.02 ^c,d^	0.35 ± 0.02 ^c^	0.129 ± 0.008 ^f^	0.44 ± 0.01 ^b^
Luteolin	0.0447 ± 0.0003 ^a^	0.0334 ± 0.0004 ^b,c^	0.0307 ± 0.0007 ^c^	0.01881 ± 0.00003 ^d^	0.0061 ± 0.0003 ^f^	0.036 ± 0.003 ^b^	0.0130 ± 0.0003 ^e^
Sum oleuropein	26 ± 1 ^b^	23.1 ± 0.3 ^b^	34 ± 1 ^a^	23.5 ± 0.3 ^b^	24.6 ± 0.2 ^b^	14.4 ± 0.2 ^c^	25.2 ± 0.2 ^b^
Sum hydroxytyrosol	0.74 ± 0.02 ^d^	0.557 ± 0.008 ^e^	0.393 ± 0.003 ^f^	0.872 ± 0.002 c	1.01 ± 0.02 ^a^	0.37 ± 0.01 ^f^	0.928 ± 0.006 ^b^
Total	42 ± 1 ^b,c^	41.0 ± 0.3 ^b,c^	49 ± 2 ^a^	37.8 ± 0.5 ^c^	40.9 ± 0.2 ^b,c^	28.1 ± 0.3 ^d^	41.26 ± 0.08 ^b,c^

**Table 5:** Antioxidant activity of olive leaf cultivars at optimum sonotrode ultrasound-assisted extraction. Different letters in the same column indicate significantly different values (*p* < 0.05).

Cultivars	DPPH	ABTS	FRAP
‘Arbequina’	43.7 ± 0.4 ^b^	29.73 ± 0.04 ^d^	49.76 ± 0.03 ^c^
‘Arbosana’	36.9 ± 0.3 ^c^	32.33 ± 0.01 ^c^	51.03 ± 0.008 ^b^
‘Changlot Real’	46.8 ± 0.2 ^a^	35.7 ± 0.1 ^a^	53.87 ± 0.04 ^a^
‘Frantoio’	41.2 ± 0.2 ^b^	28.14 ± 0.04 ^e^	38.61 ± 0.09 ^e^
‘Koroneiki’	36.7 ± 0.4 ^c^	26.92 ± 0.03 ^f^	39.27 ± 0.01 ^d^
‘Picual’	33.03 ± 0.04 ^d^	28.12 ± 0.04 ^e^	37.17 ± 0.01 ^f^
‘Sikitita’	45.83 ± 0.08 ^a^	33.24 ± 0.06 ^b^	50.7 ± 0.3 ^b^

DDPH, ABTS and FRAP are expressed as mean ± standard deviation of mg trolox equivalent g^−1^ dry matter of olive leaves.

**Table 6:** Correlation analysis of phenolic content and antioxidant activities of olive leaves extract. Different letters corresponding to the number of isomers.

	DPPH	ABTS	FRAP
Hydroxytyrosol–hexose isomer a	0.46	0.74 *	0.79 *
Oleoside	0.82 *	0.67 *	0.75 *
Hydroxytyrosol–hexose isomer b	0.21	−0.34	−0.17
Hydroxytyrosol	−0.26	−0.68 *	−0.48
Secologanoside isomer a	0.82 *	0.80 *	0.90 *
Tyrosol glucoside	0.62 *	0.28	0.39
Caffeoyl glucoside	0.10	0.37	0.66 *
Tyrosol	0.65 *	0.21	0.43
Elenolic acid glucoside isomer a	−0.26	0.04	0.33
Secologanoside isomer b	−0.70 *	−0.83 *	−0.98 *
Elenolic acid glucoside isomer b	−0.61 *	−0.66 *	−0.78 *
Oleuropein aglycon	−0.51	−0.28	−0.37
Elenolic acid glucoside isomer c	0.01	0.34	−0.02
Luteolin diglucoside	−0.11	0.05	0.40
Elenolic acid glucoside isomer d	0.00	−0.07	−0.12
Demethyloleuropein	−0.50	−0.91	−0.82 *
Hydroxyoleuropein isomer a	−0.51	−0.43	−0.74 *
Rutin	−0.27	0.01	0.25
Luteolin rutinoside	−0.18	−0.13	0.27
Luteolin glucoside isomer a	−0.05	0.13	0.43
Verbascoside	0.50	0.64 *	0.87 *
Hydroxyoleuropein isomer b	−0.18	0.46	0.51
Apigenin rutinoside	−0.28	0.41	0.45
Oleuropein diglucoside isomer a	0.38	−0.13	−0.15
Apigenin-7-glucoside	0.29	0.76 *	0.58 *
Oleuropein diglucoside isomer b	0.01	0.00	0.23
Luteolin glucoside isomer b	−0.17	−0.02	0.26
Oleuropein diglucoside isomer c	0.47	0.56 *	0.58 *
Chrysoeriol-7-O-glucoside	0.03	−0.66 *	−0.53
Luteolin glucoside isomer c	−0.78 *	−0.36	−0.21
Oleuropein isomer a	0.81 *	0.67 *	0.70 *
Oleuropein isomer b	0.57 *	0.27	0.42
Oleuropein/Oleuroside	0.71 *	0.32	0.53
Ligstroside aglycone	−0.36	−0.15	−0.22
Ligstroside	0.62	0.09	0.42
Luteolin	−0.02	0.20	0.32
Sum oleuropein	0.77 *	0.66 *	0.77 *
Sum Hydroxytyrosol	0.17	−0.40	−0.22
Total	0.82 *	0.65 *	0.70 *

Results are expressed as Pearson correlation coefficients with indicated level of significance. * Significant correlations at *p* < 0.05; DPPH = 2,2-diphenyl-1-picrylhydrazyl, FRAP = ferric reducing antioxidant power; ABTS: 2,2′-azino-di (3-ethylbenzothiazoline)-6-sulfonic acid.

**Table 7:** Minimum bactericidal concentration (MBC) values (mg mL^−1^) for olive leaf extracts against bacterial strains.

Cultivars	MBC (mg mL^−1^)				
	*S. aureus*	MRSA	*E. coli*	*S. Typhimurium*	*L. monocytogenes*
‘Arbequina’	5.6	22.5	22.4	22.5	11.3
‘Arbosana’	11.0	11.0	22.0	44.0	22.0
‘Sikitita’	5.9	11.8	11.8	23.5	11.8
‘Picual’	10.0	10.0	20.0	40.0	10.0
‘Changlot Real’	10.9	10.9	21.8	21.8	10.9
‘Frantoio’	5.5	5.5	22.0	22.0	22.0
‘Koroneiki’	11.3	11.3	22.5	45.0	22.5

## Data Availability

Data is contained within the article or Supplementary Materials.

## Supplementary Materials

The following supporting information can be downloaded at: https://www.mdpi.com/article/10.3390/antiox11030558/s1, Table S1. Analytical parameters of the proposed method. LOD: limit of detection, LOQ: limit of quantification. Table S2. Phenolic compounds quantified in olive leaf extracts by HPLC–MS expressed as mean ± standard deviation in mg g−1 matter of olive leaves. Different letters indicate significant differences among the extractions. LOQ: limit of quantification. Table S3. Quantification of phenolic compounds obtained at conventional ultrasonic assisted extraction in different olive leaf cultivars expressed as mean ± standard deviation in mg g−1 matter of olive leaves. Different letters indicate significant differences among the cultivars. Table S4. Measure of inhibition halos (mm) for olive leaf extracts against bacterial strains.
