# Supplementary material for: Comparative Extraction of Phenolic Compounds from Olive Leaves Using a Sonotrode and an Ultrasonic Bath and the Evaluation of Both Antioxidant and Antimicrobial Activity
Source: Antioxidants (Basel). 2022 Mar 15;11(3):558. doi: 10.3390/antiox11030558 (PMC8944617; doi:10.3390/antiox11030558)
Supplement: Supplementary file 1 [file antioxidants-11-00558-s001.zip › antioxidants-1615982-supplementary.pdf]

Supplementary Materials: Comparative Extraction of Phenolic Compounds from Olive Leaves Using a Sonotrode and an Ultrasonic Bath and the Evaluation of Both Antioxidant and Antimicrobial Activity

Beatriz Martín-García [1,2], Soumi De Montijo-Prieto [3], Maria Jiménez-Valera [3], Alegría Carrasco-Pancorbo [1], Alfonso Ruiz-Bravo [3], Vito Verardo [2,4,*] and Ana María Gómez-Caravaca [1,4]

[1] Department of Analytical Chemistry, Faculty of Sciences, University of Granada, Avd. Fuentenueva s/n, 18071 Granada, Spain; bea91mg@ugr.es (B.M.-G.); alegriac@ugr.es (A.C.-P.); anagomez@ugr.es (A.M.G.-C.)
[2] Department of Nutrition and Food Science, Campus of Cartuja, University of Granada, 18071 Granada, Spain
[3] Department of Microbiology, Campus of Cartuja, University of Granada, 18071 Granada, Spain; soumidemontijop@ugr.es (S.D.M.-P.); mjvalera@ugr.es (M.J.-V.); aruizbr@ugr.es (A.R.-B.)
[4] Biomedical Research Center, Institute of Nutrition and Food Technology 'José Mataix', University of Granada, Avda del Conocimiento sn., Armilla, 18100 Granada, Spain
* Correspondence: vitoverardo@ugr.es; Tel.: +34-958243864

Table S1. Analytical parameters of the method proposed. LOD: Limit of detection, LOQ: Limit of quantification.

Analyte	LOD	LOQ	Linear Range (mg L^{-1})	Calibration Curves (mg L^{-1})	r^2
Hydroxytyrosol	0.0122	0.0407	LOQ-100	y=76790x + 108991	0.9977
Tyrosol	0.0431	0.1435	LOQ-100	y= 21753x + 6114.3	0.9951
Oleuropein	0.0954	0.3179	LOQ-100	y= 6615.7x + 9603.8	0.9947
Rutin	0.2366	0.7888	LOQ-100	y= 3957.7x + 2144.5	0.9970
Luteolin-7-glucoside	0.0579	0.1929	LOQ-100	y= 16185x + 22624	0.9946
Apigenin-7-glucoside	0.0229	0.0764	LOQ-100	y= 40886x + 833.18	0.9988
Luteolin	0.0061	0.0203	LOQ-100	y= 153477x + 4221.2	0.9995

Table S2. Phenolic compounds quantified in olive leaves extracts obtain by HPLC -MS according to the experimental design expressed as mean ± standard deviation in mg g^{-1} dry matter of olive leaves. Different letters indicate significant differences among the extractions. LOQ: Limit of quantification.

Peak	Compound	SON-1	SON-2	SON-3	SON-4	SON-5	SON-6	SON-7
**1**	Hydroxytyrosol-hexose isomer a[1]	0.0027 ± 0.0007[a]	0.0028 ± 0.0003[a]	0.00026 ± 0.0008[d]	0.0016 ± 0.0003[a]	0.0028 ± 0.0002[a]	0.0024 ± 0.0001[a]	0.00253 ± 0.00006[a]
**2**	Oleoside[2]	0.45 ± 0.01[a]	0.40 ± 0.01[b]	0.2988 ± 0.0007[f]	0.32 ± 0.01[e,f]	0.362 ± 0.007[c,d]	0.3838 ± 0.0009[b,c]	0.357 ± 0.007[c,d]
**3**	Hydroxytyrosol-hexose isomer b[1]	0.62302 ± 0.00009[a]	0.70 ± 0.02[a]	0.642 ± 0.003[a]	0.66 ± 0.03[a]	0.656 ± 0.004[a]	0.65 ± 0.01[a]	0.68 ± 0.02[a]
**4**	Hydroxytyrosol[1]	0.071 ± 0.007[a]	0.0843 ± 0.0007[a]	0.067 ± 0.003[b]	0.069 ± 0.001[b]	0.077 ± 0.001[a]	0.0709 ± 0.0004[a]	0.076 ± 0.001[a]
**5**	Secologanoside isomer a[2]	2.5 ± 0.1[a]	2.2 ± 0.1[a,b]	1.893 ± 0.006[b]	1.929 ± 0.001[b]	2.10 ± 0.02[b]	2.08 ± 0.02[b]	2.1 ± 0.2[b]
**6**	Tyrosol glucoside[3]	0.181 ± 0.009[a]	0.191 ± 0.003[a]	0.167 ± 0.009[a]	0.166 ± 0.008[a]	0.1783 ± 0.0006[a]	0.187 ± 0.008[a]	0.181 ± 0.006[a]
**7**	Caffeoyl glucoside[2]	0.035 ± 0.001[b]	0.042 ± 0.004[a]	0.0234 ± 0.0003[d,e,f]	0.024 ± 0.002[d,e]	0.029 ± 0.001[b,c]	0.03011 ± 0.00003[b,c]	0.029 ± 0.001[b,c]
**8**	Tyrosol[3]	0.0052 ± 0.0002[a]	0.00587 ± 0.0008[a]	<LOQ	0.00330 ± 0.00005	0.0020 ± 0.0002[b]	0.0022 ± 0.0006[b]	0.0039 ± 0.0002[a]
**9**	Elenolic acid glucoside isomer a[2]	0.10 ± 0.02[a]	0.088 ± 0.004[a]	0.046 ± 0.009[c]	0.0486 ± 0.0007[c]	0.065 ± 0.006[b,c]	0.0717 ± 0.0004[a]	0.080 ± 0.003[a]
**10**	Secologanoside isomer b[2]	1.26 ± 0.03[a]	1.17 ± 0.03[a,b]	0.68 ± 0.01[d,e]	0.80 ± 0.01[d]	1.104 ± 0.006[b]	1.11 ± 0.02[b]	1.14 ± 0.05[a,b]
**11**	Elenolic acid glucoside isomer b[2]	0.7318 ± 0.0006[a]	0.71 ± 0.09[a,b]	0.37 ± 0.01[e]	0.421 ± 0.009[e]	0.559 ± 0.011[d]	0.57 ± 0.03[d]	0.611 ± 0.004[b,c]
**12**	Oleuropein aglycon[2]	2.29 ± 0.08[a]	2.19 ± 0.07[a]	1.070 ± 0.001[d]	1.24 ± 0.02[c]	1.98 ± 0.02[b]	1.97 ± 0.02[b]	2.03 ± 0.02[b]
**13**	Elenolic acid glucoside isomer c[2]	0.40 ± 0.01[a]	0.363 ± 0.005[a,b]	0.303 ± 0.002[c]	0.31 ± 0.02[c]	0.33 ± 0.02[b]	0.3460 ± 0.0009[b]	0.33 ± 0.01[b]
**14**	Luteolin diglucoside[4]	0.029 ± 0.003[c]	0.027 ± 0.002[c]	0.0143 ± 0.0004[e]	0.018 ± 0.002[d]	0.0215 ± 0.0002[d]	0.027 ± 0.001[c]	0.030 ± 0.002[c]
**15**	Elenolic acid glucoside isomer d[2]	0.1410 ± 0.009[a]	0.12 ± 0.01[a,b]	0.08690601[c]	0.086 ± 0.001[c]	0.112 ± 0.003[b]	0.108 ± 0.002[b]	0.111 ± 0.002[b]
**16**	Demethyloleuropein[2]	0.24 ± 0.02[d]	0.32 ± 0.02[a]	0.256 ± 0.002[c,d]	0.295 ± 0.004[a]	0.31 ± 0.01[a]	0.34 ± 0.01[a]	0.329 ± 0.004[a]
**17**	Hydroxyoleuropein isomer a[2]	0.48 ± 0.03[b]	0.59 ± 0.02[a]	0.034 ± 0.002[h]	0.032 ± 0.005[h]	0.12 ± 0.01[g]	0.151 ± 0.002[f,g]	0.167 ± 0.003[f]
**18**	Rutin[5]	0.43 ± 0.03[a]	0.45 ± 0.02[a]	0.260 ± 0.008[d,e]	0.286 ± 0.003[d]	0.375 ± 0.003[c]	0.41 ± 0.02[a]	0.43 ± 0.02[a]
**19**	Luteolin rutinoside[4]	0.067 ± 0.003[a]	0.061 ± 0.006[a,b]	0.0365 ± 0.0005[c,d]	0.043 ± 0.002[c]	0.053 ± 0.002[b]	0.0565 ± 0.0006[b]	0.060 ± 0.003[a,b]
**20**	Luteolin glucoside isomer a[4]	1.23 ± 0.03[a]	1.25 ± 0.05[a]	0.92 ± 0.01[f]	0.98 ± 0.03[e,f]	1.109 ± 0.005[c]	1.147 ± 0.008[b]	1.18 ± 0.02[a]
**21**	Verbascoside[1]	0.00180 ± 0.00006[g]	0.007208 ± 0.000006[a]	0.00537 ± 0.00006[e]	0.0050 ± 0.0003[e]	0.0057 ± 0.0001[d]	0.006520 ± 0.000002[a]	0.0066 ± 0.0003[a]
**22**	Hydroxyoleuropein isomer b[2]	0.0030 ± 0.0005[c]	0.0028 ± 0.0002[c,d]	0.0120 ± 0.0007[a]	0.0132 ± 0.0002[a]	<LOQ	<LOQ	<LOQ
**23**	Apigenin rutinoside[6]	0.0268 ± 0.0002[a]	0.0248 ± 0.0004[a]	0.0154 ± 0.0003[e,f]	0.018 ± 0.001[d,e]	0.022 ± 0.001[b]	0.023 ± 0.001[a]	0.0256 ± 0.0004[a]
**24**	Oleuropein diglucoside isomer a[2]	0.023 ± 0.003[a]	0.0270 ± 0.0009[a]	0.0151 ± 0.0003[b]	0.0192 ± 0.0001[a]	0.023 ± 0.004[a]	0.025 ± 0.001[a]	0.0229 ± 0.0009[a]
**25**	Apigenin-7-glucoside[6]	0.059 ± 0.002[a]	0.0541 ± 0.0001[a,b]	0.051 ± 0.004[a,b]	0.049 ± 0.002[a,b]	0.058 ± 0.007[a]	0.059 ± 0.004[a]	0.057 ± 0.001[a,b]
**26**	Oleuropein diglucoside isomer b[2]	0.051 ± 0.002[b]	0.069 ± 0.006[b]	0.054 ± 0.002[b]	0.0466 ± 0.0009[c]	0.053 ± 0.005[b]	0.048 ± 0.002[c]	0.035 ± 0.003[d]
**27**	Luteolin glucoside isomer b[4]	0.73 ± 0.03[a]	0.713 ± 0.006[a,b]	0.47 ± 0.01[e]	0.54 ± 0.02[d,e]	0.62 ± 0.01[c]	0.661 ± 0.002[a,b]	0.68 ± 0.02[a,b]
**28**	Oleuropein diglucoside isomer c[2]	0.102 ± 0.003[a]	0.090 ± 0.003[a]	0.094 ± 0.007[a]	0.083 ± 0.002[a]	0.103 ± 0.004[a]	0.0956 ± 0.0003[a]	0.098 ± 0.001[a]
**29**	Chrysoeriol-7-O-glucoside[4]	0.253 ± 0.008[a,b]	0.262 ± 0.002[a]	0.1988 ± 0.0006[d]	0.206 ± 0.001[d]	0.236 ± 0.001[b]	0.240 ± 0.001[a,b]	0.2493 ± 0.0002[a,b]
**30**	Luteolin glucoside isomer c[4]	0.101 ± 0.005[b,c]	0.137 ± 0.002[a]	0.10 ± 0.01[b,c]	0.119 ± 0.005[a]	0.10699 ± 0.00009[b]	0.122 ± 0.002[a]	0.137 ± 0.005[a]
**31**	Oleuropein isomer a[2]	12.24 ± 0.01[i]	19.3 ± 0.4[c,d,e]	17.1 ± 0.4[g]	17.35 ± 0.03[f,g]	18.37 ± 0.02[e,f]	19.5 ± 0.4[c,d]	19.72 ± 0.04[b,c,d]
**32**	Oleuropein isomer b[2]	0.29 ± 0.04[c]	0.43 ± 0.01[a,b]	0.47 ± 0.01[a]	0.47 ± 0.02[a]	0.436 ± 0.003[a]	0.417 ± 0.007[a,b]	0.44 ± 0.04[a]
**33**	Oleuropein/Oleuroside[2]	1.08 ± 0.07[c]	1.263 ± 0.005[a]	1.40 ± 0.04[a]	1.30 ± 0.03[a]	1.30 ± 0.04[a]	1.307 ± 0.006[a]	1.301 ± 0.004[a]
**34**	Ligstroside aglycone[2]	0.012 ± 0.004[a,b]	0.024 ± 0.002[a]	N.D.	0.008 ± 0.003[b,c]	0.014 ± 0.005[a,b]	0.0138 ± 0.0004[a,b]	0.011 ± 0.004[b,c]
**35**	Ligstroside [2]	0.28 ± 0.01[b]	0.30 ± 0.03[a]	0.34 ± 0.03[a]	0.32 ± 0.01[a]	0.289 ± 0.001[a]	0.32 ± 0.03[a]	0.294 ± 0.002[a]
**36**	Luteolin[7]	0.0240 ± 0.0009[a]	0.014 ± 0.001[c]	0.00273 ± 0.00007[g,h]	0.0042 ± 0.0003[f,g]	0.0059 ± 0.0003[e]	0.00593 ± 0.00006[e]	0.0058 ± 0.0001[e]
	Sum oleuropein	13.6 ± 0.1[i]	21.0 ± 0.4[c,d,e]	19.0 ± 0.4[f,g]	19.13 ± 0.04[f,g]	20.10 ± 0.05[e,f]	21.2 ± 0.5[b,c,d,e]	21.47 ± 0.01[b,c,d]

	Sum hydroxytyrosol	0.697 ± 0.006^{b,c}	0.79 ± 0.02^{a}	0.709 ± 0.002^{a,b,c}	0.73 ± 0.03^{a,b}	0.736 ± 0.006^{a,b}	0.72 ± 0.01^{a,b,c}		0.76 ± 0.02^{a,b}
	Total	26.5 ± 0.6^{e,f}	33.7 ± 0.8^{a}	27.5 ± 0.5^{d,e}	28.31 ± 0.06^{d}	31.20 ± 0.05^{c}	32.5 ± 0.5^{a,b,c}		33.0 ± 0.3^{a,b}
**Peak**	Compound	SON-8	SON-9	SON-10	SON-11	SON-12	SON-13	SON-14	SON-15
**1**	Hydroxytyrosol-hexose isomer a[1]	0.0023 ± 0.0002^{a}	0.0028 ± 0.0002^{a}	0.00094 ± 0.00004^{c}	0.0022 ± 0.0004^{a}	0.0013 ± 0.0001^{b}	0.0026 ± 0.0001^{a}	0.00245 ± 0.00006^{a}	0.00263 ± 0.00008^{a}
**2**	Oleoside[2]	0.350 ± 0.005^{e}	0.33 ± 0.01^{d,e}	0.240 ± 0.009^{g}	0.351 ± 0.005^{d,e}	0.297 ± 0.002^{f}	0.345 ± 0.003^{d,e}	0.356 ± 0.005^{c,d}	0.3624 ± 0.0003^{c,d}
**3**	Hydroxytyrosol-hexose isomer b[1]	0.664 ± 0.004^{a}	0.66 ± 0.03^{a}	0.58 ± 0.01^{b}	0.67 ± 0.05^{a}	0.64 ± 0.02^{a}	0.685 ± 0.05^{a}	0.685 ± 0.003^{a}	0.67 ± 0.03^{a}
**4**	Hydroxytyrosol[1]	0.079 ± 0.005^{a}	0.0756 ± 0.0004^{a}	0.0627 ± 0.0007^{c}	0.07632 ± 0.00009^{a}	0.076 ± 0.001^{a}	0.079 ± 0.006^{a}	0.080 ± 0.004^{a}	0.078 ± 0.007^{a}
**5**	Secologanoside isomer a[2]	1.92 ± 0.01^{b}	2.02 ± 0.04^{b}	1.72180602^{c}	1.911 ± 0.002^{b}	1.81 ± 0.07^{c}	1.97 ± 0.04^{b}	1.90 ± 0.05^{b}	1.88 ± 0.03^{b}
**6**	Tyrosol glucoside[3]	0.182 ± 0.001^{a}	0.1870 ± 0.0002^{a}	0.16 ± 0.01^{b}	0.1831 ± 0.0003^{a}	0.175 ± 0.006^{a}	0.18 ± 0.01^{a}	0.18 ± 0.01^{a}	0.177 ± 0.005^{a}
**7**	Caffeoyl glucoside[2]	0.02844 ± 0.00004^{c}	0.0279 ± 0.0005^{c}	0.018 ± 0.001^{f}	0.0286 ± 0.0008^{c}	0.021 ± 0.002^{e,f}	0.0311 ± 0.0005^{b,c}	0.029 ± 0.001^{c}	0.0312 ± 0.0006^{b,c}
**8**	Tyrosol[3]	0.004 ± 0.001^{a}	0.004 ± 0.001^{a}	<LOQ	0.0023 ± 0.0004^{b}	0.0019 ± 0.0003^{c}	0.0030 ± 0.0002^{a}	0.003 ± 0.001^{a}	0.0016 ± 0.0005^{c}
**9**	Elenolic acid glucoside isomer a[2]	0.072 ± 0.009^{a}	0.070 ± 0.007^{a}	0.029 ± 0.004^{e}	0.076 ± 0.001^{a}	0.047 ± 0.002^{d,e}	0.077 ± 0.007^{a}	0.078 ± 0.004^{a}	0.0755 ± 0.0004^{a}
**10**	Secologanoside isomer b[2]	1.13 ± 0.02^{b}	1.12 ± 0.05^{b}	0.60 ± 0.02^{e}	1.12 ± 0.01^{b}	0.75 ± 0.06^{d}	1.12 ± 0.02^{b}	1.08 ± 0.02^{b}	1.03 ± 0.03^{c}
**11**	Elenolic acid glucoside isomer b[2]	0.65 ± 0.02^{a,b,c}	0.56 ± 0.03^{d}	0.34 ± 0.01^{e}	0.70 ± 0.01^{a,b}	0.41 ± 0.03^{e}	0.588 ± 0.002^{c,d}	0.60 ± 0.03^{c,d}	0.595 ± 0.008^{c,d}
**12**	Oleuropein aglycon[2]	2.030 ± 0.001^{b}	1.98 ± 0.06^{b}	0.920 ± 0.004^{e}	1.983 ± 0.003^{b}	1.25 ± 0.02^{c}	1.99 ± 0.03^{b}	1.93 ± 0.01^{b}	1.96 ± 0.03^{b}
**13**	Elenolic acid glucoside isomer c[2]	0.029 ± 0.001^{e}	0.029 ± 0.006^{e}	0.289 ± 0.009^{d}	0.34 ± 0.02^{b}	0.30 ± 0.02^{c}	0.339 ± 0.008^{b}	0.314 ± 0.009^{c}	0.32 ± 0.01^{b}
**14**	Luteolin diglucoside[4]	0.168 ± 0.004^{a}	0.146 ± 0.001^{b}	0.013 ± 0.001^{f}	0.025 ± 0.002^{c}	0.0163 ± 0.0008^{e}	0.0253 ± 0.0007^{c}	0.024 ± 0.001^{c}	0.026 ± 0.001^{c}
**15**	Elenolic acid glucoside isomer d[2]	0.1073 ± 0.0006^{b}	0.108 ± 0.001^{b}	0.077 ± 0.005^{d}	0.108 ± 0.009^{b}	0.082 ± 0.003^{d}	0.116 ± 0.006^{b}	0.105 ± 0.005^{b}	0.108 ± 0.008^{b}
**16**	Demethyloleuropein[2]	0.33 ± 0.01^{a}	0.31 ± 0.01^{a,b}	0.237 ± 0.008^{d}	0.303642 ± 0.000004^{a}	0.282 ± 0.002^{b}	0.328 ± 0.009^{a}	0.341 ± 0.03^{a}	0.309 ± 0.005^{a}

**17**	Hydroxyoleuropein isomer a[2]	0.312 ± 0.002[c]	0.267 ± 0.004[d]	0.018 ± 0.006[h]	0.493 ± 0.007[b]	0.041 ± 0.003[h]	0.2220 ± 0.0008[e]	0.217 ± 0.007[e]	0.227 ± 0.009[d,e]
**18**	Rutin[5]	0.418 ± 0.007[a]	0.395 ± 0.007[b,c]	0.22 ± 0.01[e]	0.42 ± 0.01[a]	0.289 ± 0.005[d]	0.416 ± 0.006[a]	0.40 ± 0.01[a]	0.416 ± 0.006[a]
**19**	Luteolin rutinoside[4]	0.059 ± 0.002[a,b]	0.0534 ± 0.0008[b]	0.033 ± 0.003[d]	0.0596 ± 0.0001[a,b]	0.0425 ± 0.0005[c]	0.059 ± 0.001[a,b]	0.056 ± 0.002[b]	0.059 ± 0.002[a,b]
**20**	Luteolin glucoside isomer a[4]	1.10 ± 0.01[c]	1.06 ± 0.02[d,e]	0.78 ± 0.02[g]	1.12 ± 0.01[c]	0.93 ± 0.03[f]	1.18 ± 0.01[a]	1.081 ± 0.001[d]	1.09 ± 0.02[d]
**21**	Verbascoside[1]	0.00639 ± 0.00009[b]	0.0051 ± 0.0003[e]	0.0040 ± 0.0001[f]	0.00591 ± 0.00003[c]	0.0052 ± 0.0001[e]	0.00692 ± 0.00004[a]	0.0059 ± 0.0005[c]	0.0063 ± 0.0001[b]
**22**	Hydroxyoleuropein isomer b[2]	0.00027 ± 0.00002[d]	<LOQ	0.006 ± 0.002[b]	<LOQ	0.0123 ± 0.0001[a]	<LOQ	<LOQ	<LOQ
**23**	Apigenin rutinoside[6]	0.0244 ± 0.0002[a]	0.0222 ± 0.0003[b]	0.01309 ± 0.00004[f]	0.021 ± 0.001[b]	0.0189 ± 0.0005[d,e]	0.0244 ± 0.0007[a]	0.023 ± 0.001[a]	0.023 ± 0.002[a]
**24**	Oleuropein diglucoside isomer a[2]	0.023 ± 0.001[a]	0.022 ± 0.006[a]	0.008 ± 0.001[b]	0.021 ± 0.004[a]	0.019 ± 0.001[a]	0.023 ± 0.001[a]	0.022 ± 0.003[a]	0.021 ± 0.001[a]
**25**	Apigenin-7-glucoside[6]	0.059 ± 0.002[a]	0.0513 ± 0.0009[a,b]	0.044263[b]	0.057 ± 0.001[a]	0.053 ± 0.003[a,b]	0.058 ± 0.004[a]	0.052 ± 0.002[a,b]	0.0192 ± 0.0008[c]
**26**	Oleuropein diglucoside isomer b[2]	0.047 ± 0.004[c]	0.044 ± 0.006[c]	0.0412731[c]	0.046 ± 0.002[c]	0.045 ± 0.003[c]	0.056 ± 0.009[b]	0.057 ± 0.004[b]	0.21 ± 0.01[a]
**27**	Luteolin glucoside isomer b[4]	0.65 ± 0.02[b,c]	0.574 ± 0.009[c,d]	0.382 ± 0.005[f]	0.63 ± 0.01[c]	0.54 ± 0.03[d,e]	0.67 ± 0.04[a,b]	0.60 ± 0.03[c,d]	0.67 ± 0.01[a,b]
**28**	Oleuropein diglucoside isomer c[2]	0.105 ± 0.003[a]	0.093 ± 0.007[a]	0.0773 ± 0.0004[b]	0.09 ± 0.01[a]	0.09 ± 0.02[a]	0.104 ± 0.006[a]	0.113 ± 0.005[a]	0.10 ± 0.01
**29**	Chrysoeriol-7-O-glucoside[4]	0.240 ± 0.002[a,b]	0.21 ± 0.01[c,d]	0.164 ± 0.007[e]	0.231 ± 0.007[b,c]	0.21 ± 0.01[d]	0.241 ± 0.007[a,b]	0.232 ± 0.004[b]	0.244 ± 0.005[a,b]
**30**	Luteolin glucoside isomer c[4]	0.137 ± 0.003[a]	0.119 ± 0.005[a]	0.084 ± 0.009[c]	0.123 ± 0.004[a]	0.107 ± 0.006[b]	0.122 ± 0.001[a]	0.116 ± 0.003[a]	0.121 ± 0.002[a]
**31**	Oleuropein isomer a[2]	20.1 ± 0.2[a,b,c]	16.6 ± 0.4[g,h]	15.8 ± 0.3[h]	18.8 ± 0.1[d,e]	18.6 ± 0.4[d,e]	21.03 ± 0.02[a]	20.62 ± 0.02[a,b]	21.1 ± 0.4[a]
**32**	Oleuropein isomer b[2]	0.402 ± 0.009[a,b]	0.343 ± 0.008[b,c]	0.39 ± 0.02[a,b]	0.386 ± 0.004[a,b]	0.43 ± 0.01[a,b]	0.46 ± 0.01[a]	0.45 ± 0.03[a]	0.43 ± 0.04[a]
**33**	Oleuropein/Oleuroside[2]	1.20 ± 0.03[b,c]	1.14 ± 0.03[c]	1.24 ± 0.07[a]	1.14 ± 0.06[c]	1.29 ± 0.04[a]	1.29 ± 0.01[a]	1.22 ± 0.03[b,c]	1.25 ± 0.04[a]

**34**	Ligstroside aglycone[2]	0.009 ± 0.002^{b,c}	0.015 ± 0.001^{a,b}	<LOQ	0.0116 ± 0.0008^{b,c}	<LOQ	0.019 ± 0.002^{a,b}	0.018 ± 0.007^{a,b}	0.018 ± 0.002^{a,b}
**35**	Ligstroside [2]	0.28 ± 0.02^{b}	0.259 ± 0.006^{c}	0.283 ± 0.004^{b}	0.267 ± 0.009^{b}	0.298 ± 0.009^{a}	0.287 ± 0.009^{a}	0.282 ± 0.009^{b}	0.290 ± 0.006^{a}
**36**	Luteolin[7]	0.0050 ± 0.0003^{e,f}	0.0191 ± 0.0002^{b}	0.00170 ± 0.00005^{h}	0.0124 ± 0.0003^{d}	0.00373 ± 0.00007^{f,g}	0.0067 ± 0.0004^{e}	0.0060 ± 0.0001^{e}	0.0065 ± 0.0001^{e}
	Sum oleuropein	21.8 ± 0.2^{a,b,c}	18.1 ± 0.3^{g,h}	17.5 ± 0.3^{h}	20.32 ± 0.07^{e}	20.4 ± 0.4^{d,e}	22.771 ± 0.007^{a}	22.30 ± 0.02^{a,b}	22.8 ± 0.5^{a}
	Sum hydroxytyrosol	0.746 ± 0.008^{a,b}	0.73 ± 0.03^{b}	0.64 ± 0.01^{c}	0.75 ± 0.05^{a,b}	0.72 ± 0.02^{a,b,c}	0.76 ± 0.02^{a,b}	0.768 ± 0.001^{a,b}	0.75 ± 0.03^{a,b}
	Total	33.0 ± 0.3^{a,b}	28.9 ± 0.5^{d}	24.92 ± 0.06^{f}	31.82 ± 0.08^{c}	29.2 ± 0.5^{d}	34.153 ± 0.001^{a}	33.29 ± 0.4^{a,b}	34.0 ± 0.7^{a}

[1] $mg\ g^{-1}$ hydroxytyrosol. [2] $mg\ g^{-1}$ oleuropein. [3] $mg\ g^{-1}$ tyrosol. [4] $mg\ g^{-1}$ luteolin-7-glucoside. [5] $mg\ g^{-1}$ rutin, [6] $mg\ g^{-1}$ apigenin-7-glucoside, [7] $mg\ g^{-1}$ luteolin.

Table S3. Quantification of phenolic compounds obtained using conventional ultrasonic assisted extraction in different olive leaves cultivars expressed as mean ± standard deviation in $mg\ g^{-1}$ dry matter of olive leaves. Different letters indicate significant differences among the cultivars.

Peak	Compound	'Arbequina'	'Arbosana'	'Changlot Real'	'Frantoio'	'Koroneiki'	'Picual'	'Sikitita'
1	Hydroxytyrosol-hexose isomer a[1]	0.0054 ± 0.0005^{a,b}	0.0062 ± 0.0002^{a}	0.0043 ± 0.0004^{b,c}	0.0019 ± 0.0005^{d}	0.0027 ± 0.0008^{c,d}	0.0035 ± 0.0002^{c,d}	0.0067 ± 0.0003^{a}
2	Oleoside[2]	0.5748 ± 0.0001^{a}	0.48 ± 0.03^{b}	0.57 ± 0.02^{a}	0.48 ± 0.03^{b}	0.53 ± 0.04^{a,b}	0.238 ± 0.006^{c}	0.542 ± 0.005^{a,b}
3	Hydroxytyrosol-hexose isomer b[1]	0.60 ± 0.03^{c}	0.427 ± 0.003^{d}	0.325 ± 0.002^{e}	0.678 ± 0.008^{b}	0.83 ± 0.04^{a}	0.2974 ± 0.0005^{e}	0.795 ± 0.001^{a}
4	Hydroxytyrosol[1]	0.137 ± 0.002^{b}	0.116 ± 0.002^{b}	0.054 ± 0.002^{e}	0.162 ± 0.002^{a}	0.131 ± 0.001^{b}	0.103 ± 0.003^{d}	0.138 ± 0.006^{b}
5	Secologanoside isomer a[2]	3.52 ± 0.06^{a,b}	3.6 ± 0.1^{a,b}	3.9 ± 0.2^{a}	3.163 ± 0.004^{b}	3.8 ± 0.1^{a}	2.19 ± 0.03^{c}	3.74 ± 0.1^{a}
6	Tyrosol glucoside[3]	0.16 ± 0.04^{c}	0.081 ± 0.005^{d}	0.17 ± 0.01^{c}	0.106 ± 0.005^{c}	0.29 ± 0.02^{b}	0.020 ± 0.001^{d}	0.418 ± 0.003^{a}
7	Caffeoyl glucoside[2]	0.27 ± 0.08^{a}	0.29 ± 0.02^{a}	0.127 ± 0.007^{b}	0.01287 ± 0.00009^{c}	0.030 ± 0.001^{c}	0.016 ± 0.002^{c}	0.038 ± 0.007^{c}
8	Tyrosol[3]	0.030 ± 0.002^{b}	0.0181 ± 0.0009^{c}	0.0201 ± 0.0008^{c}	0.01826 ± 0.00009^{c}	0.0163 ± 0.0006^{c}	0.017 ± 0.001^{c}	0.037 ± 0.001^{a}
9	Elenolic acid glucoside isomer a[2]	0.147 ± 0.007^{b}	0.23 ± 0.02^{a}	0.05 ± 0.01^{c}	0.053 ± 0.007^{c}	0.13 ± 0.01^{b}	0.065 ± 0.004^{c}	0.048 ± 0.009^{c}
10	Secologanoside isomer b[2]	0.87 ± 0.02^{c}	0.82 ± 0.04^{c}	0.57 ± 0.02^{d}	1.91 ± 0.03^{a,b}	1.7 ± 0.1^{b}	2.01 ± 0.03^{a}	0.75 ± 0.01^{c,d}
11	Elenolic acid glucoside isomer b[2]	0.70 ± 0.02^{b}	0.87 ± 0.04^{a}	0.64 ± 0.03^{b}	0.87 ± 0.02^{a}	0.87 ± 0.08^{a}	0.75 ± 0.01^{a,b}	0.636 ± 0.008^{b}
12	Oleuropein aglycon[2]	0.554 ± 0.001^{e}	1.32 ± 0.01^{b}	1.25 ± 0.05^{b,c}	0.94 ± 0.04^{d}	2.6 ± 0.02^{a}	1.16 ± 0.03^{c}	0.83 ± 0.01^{d}
13	Elenolic acid glucoside isomer c[2]	0.25 ± 0.03^{e}	0.37 ± 0.05^{d}	0.96 ± 0.02^{a}	0.38 ± 0.03^{d}	0.60 ± 0.02^{c}	0.819 ± 0.008^{b}	0.67 ± 0.02^{c}
14	Luteolin diglucoside[4]	0.080 ± 0.0001^{b}	0.102 ± 0.007^{a}	0.025 ± 0.004^{d}	0.031 ± 0.002^{c,d}	0.049 ± 0.007^{c}	0.047 ± 0.006^{c}	0.048 ± 0.001^{c}
15	Elenolic acid glucoside isomer d[2]	0.08 ± 0.01^{c}	0.09 ± 0.01^{c}	0.074 ± 0.009^{c}	0.102 ± 0.006^{b,c}	0.143 ± 0.005^{b}	0.11 ± 0.02^{b,c}	0.186 ± 0.005^{a}
16	Demethyloleuropein[2]	0.46 ± 0.03^{b}	0.225 ± 0.008^{c}	0.18 ± 0.02^{c,d}	0.532 ± 0.008^{a}	0.47 ± 0.01^{a,b}	0.48 ± 0.03^{a,b}	0.14 ± 0.01^{d}

**17**	Hydroxyoleuropein isomer a[2]	0.072 ± 0.004[c]	0.082 ± 0.004[c]	0.1755 ± 0.0002[b]	0.202 ± 0.006[b]	0.17 ± 0.03[b]	0.3133 ± 0.0007[a]	0.106 ± 0.008[c]
**18**	Rutin[5]	0.53 ± 0.03[b]	1.37 ± 0.07[a]	0.21 ± 0.01[d]	0.40 ± 0.01[c]	0.61 ± 0.04[b]	0.1682 ± 0.0009[d]	0.374 ± 0.004[c]
**19**	Luteolin rutinoside[4]	0.136 ± 0.003[a]	0.14 ± 0.01[a]	0.023 ± 0.003[e]	0.0475 ± 0.0008[c,d]	0.09 ± 0.01[b]	0.0525 ± 0.0005[c,d]	0.073 ± 0.004[b,c]
**20**	Luteolin glucoside isomer a[4]	2.38 ± 0.09[b]	2.9 ± 0.1[a]	1.39 ± 0.06[d]	1.84 ± 0.06[c]	1.89 ± 0.07[c]	1.42 ± 0.03[d]	1.78 ± 0.08[c]
**21**	Verbascoside[1]	0.023 ± 0.001[a]	0.0204 ± 0.0002[a]	0.019 ± 0.002[a]	0.0075 ± 0.0001[b]	0.0091 ± 0.0006[b]	0.0101 ± 0.0006[b]	0.018 ± 0.004[a]
**22**	Hydroxyoleuropein isomer b[2]	0.025 ± 0.002[c]	0.090 ± 0.02[a,b]	0.13 ± 0.001[a]	0.06 ± 0.01[b]	0.0032 ± 0.0002[d]	0.079 ± 0.005[a]	0.064 ± 0.002[b]
**23**	Apigenin rutinoside[6]	0.0329 ± 0.0002[b,c]	0.062 ± 0.001[a]	0.040 ± 0.004[b]	0.024 ± 0.002[c]	0.038 ± 0.001[b]	0.038 ± 0.005[b]	0.037 ± 0.004[b]
**24**	Oleuropein diglucoside isomer a[2]	0.0357 ± 0.0001[b]	0.042 ± 0.004[b]	0.035 ± 0.003[b]	0.04 ± 0.01[b]	0.055 ± 0.001[a]	0.03 ± 0.01[b]	0.055 ± 0.005[a]
**25**	Apigenin-7-glucoside[6]	0.099 ± 0.005[c]	0.30 ± 0.01[a]	0.287 ± 0.009[a]	0.172 ± 0.002[b]	0.107 ± 0.003[c]	0.116 ± 0.003[b,c]	0.1409 ± 0.0002[b]
**26**	Oleuropein diglucoside isomer b[2]	0.043 ± 0.002[b]	0.0562 ± 0.0003[b]	0.036 ± 0.003[b]	0.040 ± 0.008[b]	0.09 ± 0.01[a]	0.034 ± 0.003[b]	0.0769 ± 0.0006[a]
**27**	Luteolin glucoside isomer b[4]	1.29 ± 0.01[a,b]	1.45 ± 0.09[a]	0.655 ± 0.003[e]	0.85 ± 0.02[d,e]	0.84 ± 0.06[d,e]	0.92 ± 0.04[c,d]	1.12 ± 0.08[b,c]
**28**	Oleuropein diglucoside isomer c[2]	0.104 ± 0.004[b]	0.12 ± 0.02[a,b]	0.14 ± 0.01[a,b]	0.046 ± 0.003[c]	0.147 ± 0.002[a]	0.05 ± 0.01[c]	0.158 ± 0.002[a]
**29**	Chrysoeriol-7-O-glucoside[4]	0.39 ± 0.01[a]	0.23 ± 0.01[c]	0.22 ± 0.01[c]	0.37 ± 0.01[a,b]	0.35 ± 0.03[a,b]	0.29 ± 0.03[b]	0.382 ± 0.009[a]
**30**	Luteolin glucoside isomer c[4]	0.213 ± 0.006[b,c]	0.27 ± 0.02[a]	0.119 ± 0.002[d]	0.094 ± 0.003[d]	0.232 ± 0.002[a,b]	0.26 ± 0.02[a]	0.173 ± 0.005[c]
**31**	Oleuropein isomer a[2]	24.7 ± 0.2[b]	21.9 ± 0.1[b,c]	33 ± 2[a]	22 ± 1[b,c]	20.7 ± 0.3[c]	15.24 ± 0.07[d]	24.1 ± 0.3[b,c]
**32**	Oleuropein isomer b[2]	0.58 ± 0.01[a,b]	0.52 ± 0.04[b,c]	0.8 ± 0.1[a]	0.55 ± 0.02[a,b]	0.640 ± 0.002[a,b]	0.318 ± 0.001[c]	0.646 ± 0.003[a,b]
**33**	Oleuropein/Oleuroside[2]	2.09 ± 0.06[a,b]	1.79 ± 0.03[b]	2.1 ± 0.1[a,b]	1.97 ± 0.03[b]	2.2 ± 0.2[a]	0.8 ± 0.1[c]	2.16 ± 0.08[a]
**34**	Ligstroside aglycone[2]	0.226 ± 0.001[c]	0.28 ± 0.01[b]	0.143 ± 0.001[d]	0.292 ± 0.003[b]	0.038 ± 0.001[e]	0.35 ± 0.03[a]	0.187 ± 0.006[c,d]
**35**	Ligstroside [2]	0.88 ± 0.05[a]	0.473 ± 0.002[c]	0.96 ± 0.02[a]	0.416 ± 0.007[c]	0.49 ± 0.03[c]	0.19 ± 0.03[d]	0.71 ± 0.05[b]
**36**	Luteolin[7]	0.032 ± 0.002[b]	0.035 ± 0.004[b]	0.0104 ± 0.0004[c,d]	0.0191 ± 0.0005[b]	0.0053 ± 0.0007[d]	0.0452 ± 0.0006[a]	0.0157 ± 0.0004[b,c]
	Sum oleuropein	27.4 ± 0.2[b]	24.21 ± 0.05[b]	36 ± 2[a]	24.9 ± 0.9[b]	23.5 ± 0.5[b]	16.34 ± 0.03[c]	26.5 ± 0.5[b]
	Sum hydroxytyrosol	0.74 ± 0.02[c]	0.549 ± 0.001[d]	0.383 ± 0.003[e]	0.842 ± 0.007[b]	0.97 ± 0.04[a]	0.404 ± 0.004[e]	0.940 ± 0.005[a]
	Total	42.5 ± 0.5[b]	41.1 ± 0.8[b]	49 ± 2[a]	39 ± 1[b]	41 ± 1[b]	29.0 ± 0.3[c]	41.0 ± 0.8[b]

[1] mg g^{-1} hydroxytyrosol. [2] mg g^{-1} oleuropein. [3] mg g^{-1} tyrosol. [4] mg g^{-1} luteolin-7-glucoside. [5] mg g^{-1} rutin, [6] mg g^{-1} apigenin-7-glucoside, [7] mg g^{-1} luteolin.

Table S4. Measure of inhibition halos (mm) for olive leaf extracts against bacterial strains.

	S. aureus	MRSA	*E. coli*	*S. Typhimurium*	*L. monocytogenes*
'Arbequina'	17.0 (±1.0)	17.3 (±0.6)	10.3 (±0.6)	10.3 (±0.6)	10.7 (±0.6)
'Arbosana'	16.7 (±1.5)	17.7 (±0.6)	10.3 (±0.6)	10.3 (±0.6)	10.0 (±0.0)
'Sikitita'	16.7 (±1.2)	18.3 (±0.6)	10.3 (±0.6)	10.3 (±0.6)	11.7 (±0.6)
'Picual'	14.3 (±0.6)	14.7 (±0.6)	10.3 (±0.6)	10.0 (±0.0)	10.3 (±0.6)
'Changlot Real'	19.7 (±0.6)	20.3 (±0.6)	10.7 (±0.6)	10.3 (±0.0)	11.7 (±0.6)
'Frantoio'	18.3 (±1.5)	19.0 (±1.0)	10.0 (±0.0)	10.3 (±0.6)	12.3 (±0.6)
'Koroneiki'	18.0 (±1.0)	19.0 (±1.0)	10.0 (±0.0)	10.0 (±0.0)	11.3 (±0.6)
'Ciprofloxacin'	20.0 (±0.6)	25.0 (±0.6)	42.0 (±1.2)	40.0 (± 0.6)	15.4 (± 0.4)

S. aureus: Staphylococcus aureus; MRSA: methicillin-resistant Staphylococcus aureus; E. coli: Escherichia coli; S. Typhimurium: Salmonella enterica serovar Typhimurium; L. monocytogenes: Listeria monocytogenes. Antimicrobial agent: ciprofloxacin 2 mg mL^{-1} for MRSA, 0.1 mg mL^{-1} for L. monocytogenes, 0.01 mg mL^{-1} for the rest of bacteria. Each value is expressed as means ± standard deviation (n = 3).
